# Time to Change for Mental Health and Well-being via Virtual Professional Coaching: Longitudinal Observational Study

**DOI:** 10.2196/27774

**Published:** 2021-07-05

**Authors:** Alexis M Jeannotte, Derek M Hutchinson, Gabriella R Kellerman

**Affiliations:** 1 BetterUp, Inc San Francisco, CA United States

**Keywords:** professional coaching, virtual coaching, mental health, psychological well-being, stress, resilience, life satisfaction, longitudinal, intervention, well-being, satisfaction, coach, observational

## Abstract

**Background:**

Optimal mental health yields many benefits and reduced costs to employees and organizations; however, the workplace introduces challenges to building and maintaining mental health that affect well-being. Although many organizations have introduced programming to aid employee mental health and well-being, the uptake and effectiveness of these efforts vary. One barrier to developing more effective interventions is a lack of understanding about how to improve well-being over time. This study examined not only whether employer-provided coaching is an effective strategy to improve mental health and well-being in employees but also how this intervention changes well-being in stages over time.

**Objective:**

The goal of this study was to determine whether BetterUp, a longitudinal one-on-one virtual coaching intervention, improves components of mental health and psychological well-being, and whether the magnitude of changes vary in stages over time. This is the first research study to evaluate the effectiveness of professional coaching through three repeated assessments, moving beyond a pre-post intervention design. The outcomes of this study will enable coaches and employers to design more targeted interventions by outlining when to expect maximal growth in specific outcomes throughout the coaching engagement.

**Methods:**

Three identical assessments were completed by 391 users of BetterUp: prior to the start of coaching, after approximately 3-4 months of coaching, and again after 6-7 months of coaching. Three scales were used to evaluate psychological and behavioral dimensions that support management of mental health: stress management, resilience, and life satisfaction. Six additional scales were used to assess psychological well-being: emotional regulation, prospection ability, finding purpose and meaning, self-awareness, self-efficacy, and social connection.

**Results:**

Using mixed-effects modeling, varying rates of change were observed in several dimensions of mental health and psychological well-being. Initial rapid improvements in the first half of the intervention, followed by slower growth in the second half of the intervention were found for prospection ability, self-awareness, self-efficacy, social connection, emotional regulation, and a reduction in stress (range of unstandardized β values for each assessment: .10-.19). Life satisfaction improved continuously throughout the full intervention period (β=.13). Finding purpose in meaning at work and building resilience both grew continuously throughout the coaching intervention, but larger gains were experienced in the second half of the intervention (β=.08-.18), requiring the full length of the intervention to realize maximal growth.

**Conclusions:**

The results demonstrate the effectiveness of BetterUp virtual one-on-one coaching to improve psychological well-being, while mitigating threats to mental health such as excessive and prolonged stress, low resilience, and poor satisfaction with life. The improvements across the collection of outcomes were time-dependent, and provide important insights to users and practitioners about how and when to expect maximal improvements in a range of interrelated personal and professional outcomes.

## Introduction

### Background

In recent years, the cost of mental health to organizations worldwide has grown dramatically, with poor mental health becoming one of the leading risks to worker well-being and safety [1]. In the workplace, poor mental health, and its clinical counterpart mental illness, can present significant challenges to individual, team, and organizational functioning, with approximately 19% of working adults in developed countries suffering from a behavioral health disorder [2,3] and almost US $1 trillion dollars in lost productivity to the global economy as a result of depression and anxiety alone [4]. Importantly, many of the symptoms associated with poor mental health do not rise to the level of a clinical diagnosis. Even without reaching this threshold, these symptoms can still be debilitating, or at least disruptive, by impacting the daily lives and professional contributions of those affected [5-9].

Just as poor mental health can cause personal and professional problems, positive psychological well-being at work is associated with a range of beneficial life outcomes. Such benefits include greater happiness, improved health, and increased longevity [10-14]. Improving mental health can also yield benefits to organizations that transcend personal well-being, which include higher levels of job satisfaction, greater organizational commitment, increased work effort, lower employee turnover, lower rates of absenteeism, and fewer workplace accidents [15]. Boehm and Lyobumirsky [16] suggested that happier employees display more citizenship behaviors in the workplace, are better performers, and earn higher salaries, demonstrating tangible benefits for themselves, their colleagues, and their employers.

A multitude of employer-provided interventions have proven to be beneficial in helping employees to both address poor mental health and improve psychological well-being. Such programs include workplace wellness programs, employee assistance programs, mentoring programs, and employee affinity groups [17-22]. The effectiveness of such interventions varies, with usage and efficacy depending on employee uptake, perceptions of stigma around seeking treatment or support, as well as explicit or implicit signals from organizational leadership [22-27]. However, almost nothing is known about how these interventions differentially impact components of well-being over time. This knowledge gap prevents deeper understanding of how these interventions drive change at a level that can ultimately inform the design of even more effective, targeted, and personalized offerings.

In this study, we examined the longitudinal effectiveness of BetterUp, a virtual one-on-one professional coaching platform provided by employers to improve employee mental health and well-being. Professional coaching as an employee benefit is growing in popularity among employers with increasing evidence about its effectiveness to help develop a range of personal and professional mindsets and behaviors to include those that support psychological well-being [28-30]. In particular, BetterUp may overcome the limitations of other employer-provided resources to improve mental health by enabling support that is personalized to the employee, adapts as their needs evolve, and provides access to a coach when and where they need it— whether that be at work or outside of work—avoiding potential stigma or perceptions that seeking support could be viewed negatively by the employer.

We investigated the longitudinal and sequential impact of BetterUp on a set of positive mental health outcomes and related dimensions that help mitigate poor mental health. As shown in Table 1, the measured outcomes were aligned to six dimensions of positive psychological well-being [31] and three dimensions that help to bolster positive mental health, while mitigating negative mental health: stress management [32], resilience [33], and life satisfaction [34]. Critically, maintenance or improvement of each of these dimensions can directly impact personal and professional success [35].

**Table 1:** Alignment of study outcomes and associated measurement scales to dimensions from prior models of positive mental health and psychological well-being.

Positive mental health and psychological well-being dimensions		BetterUp dimensions			
Dimension	Definition	Dimension	Reliability (Cronbach α)	Items (n)	Sample item
Autonomy [31]	Self-determining and independent; regulates behavior from within	Emotional regulation	.84	3	I have effective strategies for maintaining control of my emotions
Environmental mastery [31]	Sense of competence in managing the context around them	Self-efficacy	.84	3	I believe I can achieve the things that I really want in life
Self-acceptance [31]	Positive attitude toward the self, including good and bad aspects	Self- awareness	.70	4	I have a good sense of the things in life that bring me joy
Purpose in life [31]	Holds beliefs that give life purpose; feels meaning in present and past life	Purpose and meaning	.92	3	The work I do makes an impact
Personal growth [31]	Motivation for continued growth and development	Prospection	.87	3	I think about how to make the most out of my future
Positive relations with others [31]	Satisfying and trusting relationships, understands give and take	Social connection	.80	3	I regularly interact with people who give me support and encouragement
Stress [32]	Experience of tension as a result of personal or work circumstances	Stress	.89	3	I experience a great deal of tension in my daily life
Resilience [33]	Cope, recover, and grow from challenging circumstances	Resilience	.88	3	I recover quickly after stressful experiences
Life satisfaction [34]	Feeling of fulfillment in life	Life satisfaction	.84	2	To what extent are the things you do in your life worthwhile?

### Objectives

The goal of this study was to determine whether BetterUp, a longitudinal one-on-one virtual coaching intervention, improved components of mental health and psychological well-being, and whether the magnitude of changes varied over time. Toward this end, we examined the set of outcomes at three different time points to map out the developmental timeline of the professional coaching intervention provided by BetterUp. We surveyed identical outcomes before coaching began, at baseline, and then again at two subsequent time points after coaching began to understand if coaching impacts specific mental health and well-being outcomes, when significant changes occur, and whether improvements are maintained over time.

To our knowledge, this is the first time the effectiveness of professional coaching has been studied with more than two time points (ie, a single baseline and one postcoaching assessment). In particular, our novel use of multiple, repeated assessments to understand the length of time it takes to maximize the benefits of professional coaching for employees enables gaining unique insight into the timeline to slow or prevent a decline in mental health (eg, increasing stress) or bolster positive psychological well-being in individuals. The outcomes of this study will enable coaches and employers to design more targeted interventions by determining when to expect maximal growth in specific outcomes throughout the coaching engagement.

## Methods

### Study Design and Participants

This study used a longitudinal, observational within-subjects design to evaluate individual changes in the personal and professional mental health and well-being outcomes of BetterUp users. Although users are continuously engaged on the BetterUp platform, we a priori set the data collection window for approximately 1 year to allow sufficient time for users to complete the program. For this study, program completion was defined as completing at least eight coaching sessions, an initial baseline assessment, additional assessments every 3-4 months across two time points, and remaining active on the platform for approximately 6 months. Participation on the platform was voluntary and individuals had the option to stop using the services at any time.

The use of human subject data was reviewed and determined to be exempt by the Ethical and Independent Review Services Institutional Review Board.

### Intervention

**Figure 1:**
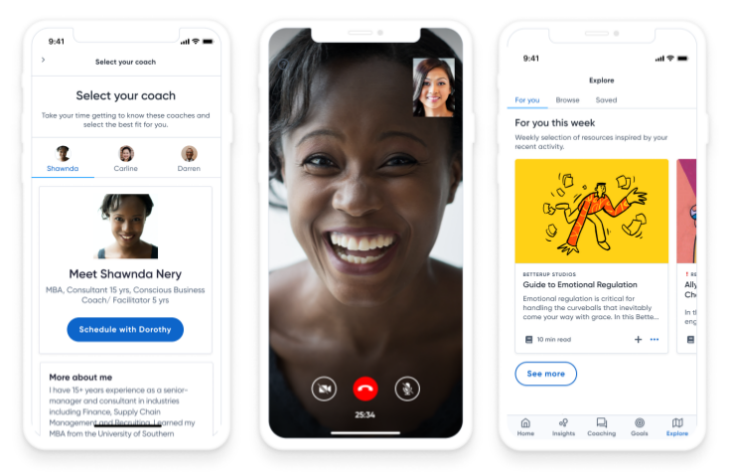
BetterUp Platform. The BetterUp virtual coaching platform offers users an algorithm-driven coach-selection process (left), coaching sessions primarily via video (middle), and resources for further personal development resulting from hybrid coach and algorithm recommendations (right).

BetterUp provides one-on-one virtual coaching to support an individual’s personal and professional development. The platform provides coachees access to over 2000 coaches with the coach/coachee fit optimized by a set of algorithms that make recommendations based on the coachee’s personal and professional characteristics, motivations, and interests (Figure 1). Each coaching session is conducted primarily via video with a professionally certified coach and lasts approximately 30-45 minutes (Figure 1). Although the majority of the communications occur via video, the platform also allows for text messaging and phone communications.

Users of BetterUp complete an assessment of their mental health, well-being, and professional attributes and goals prior to starting the program. They complete these assessments again at approximately 3 to 4-month intervals to evaluate progress. Importantly, no demographic information is collected at any time. The user community is global, coming from countries with varying information privacy and protections requirements. BetterUp chooses not to collect demographic information to maintain privacy and protect the data of its users regardless of their geographical location.

Once the longitudinal coaching engagement is initiated, coaches select and push resources to coachees to further their development outside the coaching session. An algorithm recommends resources to the coach based on the topic of a coaching session, which include guided or self-guided exercises, readings, and audio or video content (Figure 1). Additionally, the platform provides the coachee a set of features to personalize their experience, to include setting and tracking goals, receiving nudges to support behavior change, and joining group-based sessions.

The type of coaching offered by BetterUp adheres to a traditional model of coaching; that is, a “one-to-one learning and development intervention that uses a collaborative, reflective, goal-focused relationship to achieve professional outcomes that are valued by the coachee” [28]. The defining practices of the coaches are the activities of listening, asking questions, empowering, and encouraging goal-setting, in contrast to providing specific recommendations or advice [36-38]. During a typical session, the coachee will take the lead in defining discussion topics, allowing them control over their own development and growth at a comfortable pace [39]. Furthermore, coaching is a practice distinct from clinical care such as therapy, in that it is future-focused, rather than an exploration and understanding of past experiences [40,41]. Coaching provides a resource to support the development of beneficial mindsets and sustained behavior change, which are necessary to mitigate negative mental health and support positive well-being to potentially protect against worsening symptoms and the need for clinical care.

### Measure Development and Validation

The primary outcomes used to evaluate individual growth align with a theory-based conception of positive mental health, as distinct from mental illness, and well-being operationalized in a set of self-report survey measures developed by Carol Ryff and colleagues [31,42]. This model defines six dimensions of well-being: autonomy, environmental mastery, personal growth, positive relations with others, purpose in life, and self-acceptance. Further development of this model of mental health identified three other important dimensions of psychological well-being that both bolster positive mental health and buffer against negative or declining mental health: stress management [32], resilience [33], and life satisfaction [34].

The existing scales to evaluate these nine dimensions had to be adapted to be used as part of BetterUp for three primary reasons: (1) the original short versions of the existing scales had poor psychometric properties in some cases, and the 7-item version was too lengthy for commercial purposes [43,44]; (2) the scales and items needed to be framed to assess how work and the workplace shaped employee perceptions of these dimensions; and (3) BetterUp requires commercial usage of the scales and does not qualify exclusively as use for research purposes. As a result, the set of scales that measure these nine dimensions were newly developed for BetterUp and validated in a large multiphase study performed by the coaching provider [45]. The development and validation of these scales are briefly described here, with supporting documentation provided in Multimedia Appendix 1.

Initial items were developed using related measures from the literature and interviews with stakeholders. The full set of items was validated using a large sample of working professionals (N=1030) recruited from the online Amazon Mechanical Turk platform. All initial items were evaluated using various classical test theory item statistics, which included means, SD, skewness, kurtosis, interitem correlations, item-total correlations, and Cronbach α (>.70). Each scale was trimmed down from 3-10 items to 1-4 items, and all scales used in this study were composed of 3-4 items. Final scale reliabilities are reported below for each of the selected scales used in this study.

Convergent validity was determined using measures previously validated in the leadership and well-being literature. For the selected measures in this study, these included the Authentic Leadership Questionnaire [46], Psychological Capital [47], PERMA (positive emotion, engagement, relationships, meaning, accomplishments) [48], self-regulation [49], life satisfaction [50], and meaning and purpose [51]. In unpublished work utilizing a cohort of approximately 1000 US professionals, we also examined the convergent validity of a subset of the measures in this study with a brief screening tool for depression and anxiety, the Patient Health Questionnaire 4 (PHQ-4) [52,53]. Specifically, we found significant negative correlations between PHQ-4 and life satisfaction (*r_t_* –0.390, *P*<.001), prospection (*r_t_* –0.201, *P*<.001), purpose and meaning (*r_t_* –0.192, *P*<.001), resilience (*r_t_* –0.326, *P*<.001), and social connection (*r_t_* –0.326, *P*<.001). Additionally, other outcomes not measured in this study also had significant negative relationships with PHQ-4, including happiness and optimism (*r_t_* –0.422 and –0.295, respectively; *P*<.001).

Multivariate analyses of variance (ANOVAs) were performed to detect differences across managerial status (ie, people leader or individual contributor), gender, and ethnicity on each scale. All variables demonstrated linearity and acceptable univariate normality. Finally, a test-retest assessment was performed at 1 month to assess the reliability of each outcome over time. The test-retest correlations were moderate to high at 1 month (*r*_s_=0.64-0.85), reflecting acceptable and minimal change over time in a noncoached sample.

Reliabilities and example items for each of the nine scales are listed in Table 1. Except where noted, each outcome was measured using a set of three items that were rated by participants for their level of agreement using a 1-5 Likert-type scale.

### Statistical Analyses

The within-subject means of item-level responses were determined for each of the outcomes. The means and SDs within the sampled population are reported for each outcome. The Pearson product-moment correlation coefficient was determined for each outcome pairing along with the 95% CI around the coefficient.

To evaluate the change in the outcome measures, our repeated-measures data were evaluated through a series of linear mixed-effect multilevel models using the lme4 package in R [54]. Multilevel modeling allows for the evaluation of both fixed and random effects when there exists substantial nonindependence in observations. This technique represents a more robust statistical approach than repeated-measures ANOVA [55]. All analyses included random intercepts for subject ID to control for the correlation between repeated measurement of the same subjects. The sole predictor in each model was the assessment number that represents the time point during the longitudinal coaching journey that each assessment was administered. Specifically, the baseline (before beginning coaching) assessment was denoted as T1, the second assessment was denoted as T2, and the third and final assessment was denoted as T3.

We first tested if there were significant differences in growth across study measures based on the time point (ie, T1, T2, T3). To evaluate this effect, we constructed a multilevel model assessing the cross-level interaction between a factor composed of our measures and the time point. We report an ANOVA summary statistic based on the fixed effects of the model using the Type III Kenward-Rogers method of approximation produced by the lmerTest package in R [56]. For this specific analysis, our stress measure was reverse-coded to eliminate the chance that our results would be overly skewed by this sole variable decreasing over time, while all others increased over time.

To visualize the change across time, we calculated the group mean–centered effect for each outcome. Scores at each time point were calculated by subtracting an individual’s mean across all three time points from their score at each time point. Calculation of the group mean–centered effect allows for a clearer evaluation of the pattern of growth for each coaching outcome by removing variance between individuals.

To examine the magnitude of change of each outcome over time, we calculated the effect size (Cohen *d*) of each variable for each time period (T1 to T2 and T2 to T3). We then calculated the growth ratio by dividing the effect size from T1 to T2 by the effect size from T2 to T3 to derive an understanding of relative growth across the full coaching engagement. We then applied statistical testing to assess the differences in magnitude of growth between the two time periods. We performed a post hoc test for each outcome measure using the Ken-Roger method of approximation and the Tukey adjustment.

All statistics are reported with a threshold of *P*<.05 considered for statistical significance; where noted, corrections for multiple comparisons were used. The source code to replicate the statistical analyses is included in Multimedia Appendix 2. The datasets generated and analyzed during this study are available from the corresponding author on reasonable request.

## Results

### Cohort Characteristics

We included BetterUp users who completed the program between July 15, 2019 and June 24, 2020. The average completion time of the program was 184 days, or approximately 6 months. The exact length of the program varied, as the coachees had flexibility in how often they scheduled coaching sessions and their personal development goals. We set broad inclusion criteria, as described in the Methods section, and had no additional individual exclusions. The final cohort consisted of 391 participants, representing 33% of those who started the program and met the inclusion criteria. A series of independent-sample *t* tests were performed on the initial baseline (ie, T1) assessment across all study variables. No significant differences were found between those who opted into completing follow-up assessments and those who did not. Additionally, of those who did not complete the program, 49% dropped out between T1 and T2, while 51% dropped out between T2 and T3. Data from all 391 participants were used in mixed linear effects modeling.

The BetterUp platform does not collect standard demographic data; therefore, we do not report any results that consider age, gender, or other personal demographic variables. Participants were native English speakers, primarily working and living in the United States. These individuals represented a range of organizations and industries, including professional services, financial services and banking, technology, hospitality, retail, and manufacturing. Among the 391 participants, 75% (n=293) self-identified as managers, while 25% (n=98) identified as individual contributors. This cohort reflects a varied cross-section of workers in the United States today but was not selected to match the exact demographics of this working population.

### Multilevel Modeling of Outcomes

A summary of the means (SD) and intercorrelations along with their 95% CIs for all study measures are reported in Table 2; intercorrelations were all significant at *P*<.001. Each measure was aggregated within each individual across all three time points.

The average number of days from T1 to T2 was 97 (SD 42) and the average number of days from T2 to T3 was 87 (SD 49). The variability between assessment points reflected individual variability in schedules and availability to complete coaching sessions, a feature of studying coaching in an applied setting with busy, working professionals.

We first tested if there were significant differences in growth across study measures based on the time point the assessment was completed. As reported in Table 3, we found a significant interaction between the measure factor and the time point. This finding suggests that growth across the assessment time points depends on the specific measure.

The mixed-effect models across all coaching outcome measures are summarized with unstandardized β weights in Table 4. For all models, the intraclass correlation coefficient was greater than 0.50, which suggested substantial variance between individuals across all outcomes. For all variables, there was a significant effect of assessment number on each outcome variable. Our results indicate significant growth during the coaching engagement for the entire set of outcomes (all *P*<.001). The marginal R^2^ values represent the proportion of variance explained by the assessment time point [57].

**Figure 2:**
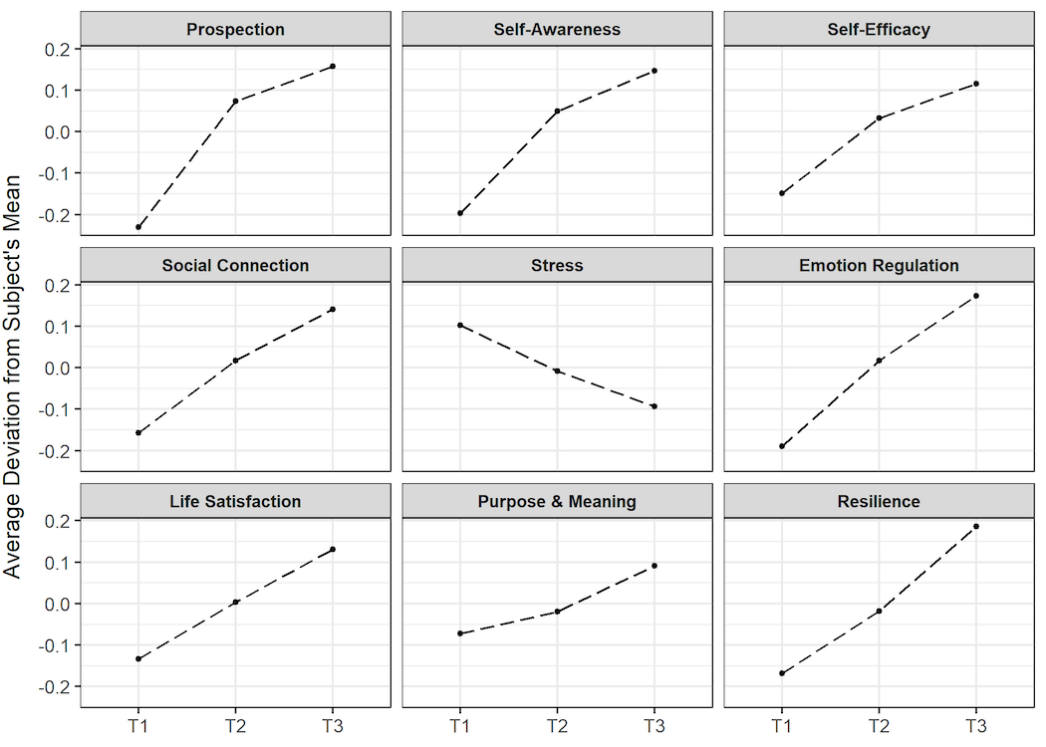
Outcome-level growth trajectories over time. Scores for each outcome measure are shown as group mean–centered effects at baseline (T1), during the intervention (T2), and at the end of the intervention (T3).

The group mean–centered effects for each variable across time are shown in Figure 2. By removing the variance between individuals, the pattern of growth for each coaching outcome is more clearly observed.

**Table 2:** Means and correlations of outcome measures with confidence intervals.^a^

Variable			Emotional regulation	Life satisfaction	Prospection	Purpose and meaning	Resilience	Self-awareness	Self-efficacy	Stress
**Emotional regulation: mean 3.72 (SD 0.64)**										
	*r*	1		0.31	0.19	0.18	0.46	0.28	0.26	–0.30
	95% CI	—^b^		0.21 to 0.39	0.09 to 0.28	0.08 to 0.27	0.38 to 0.54	0.19 to 0.37	0.17 to 0.35	–0.39 to –0.21
**Life satisfaction: mean 4.08 (SD 0.58)**										
	*r*	0.31		1	0.44	0.50	0.39	0.56	0.46	–0.51
	95% CI	0.21 to 0.39		—	0.36 to 0.52	0.42 to 0.57	0.30 to 0.47	0.48 to 0.62	0.38 to 0.54	–0.58 to –0.44
**Prospection: mean 3.91 (SD 0.59)**										
	*r*	0.19		0.44	1	0.32	0.29	0.64	0.44	–0.27
	95% CI	0.09 to 0.28		0.36 to 0.52	—	0.23 to 0.40	0.20 to 0.38	0.58 to 0.70	0.36 to 0.52	–0.36 to –0.18
**Purpose and meaning: mean 4.16 (0.60)**										
	*r*	0.18		0.50	0.32	1	0.26	0.37	0.37	–0.26
	95% CI	0.08 to 0.27		0.42 to 0.57	0.23 to 0.40	—	0.17 to 0.35	0.28 to 0.46	0.28 to 0.45	–0.35 to –0.17
**Resilience: mean 3.75 (SD 0.63)**										
	*r*	0.46		0.39	0.29	0.26	1	0.36	0.43	–0.43
	95% CI	0.38 to 0.54		0.30 to 0.47	0.20 to 0.38	0.17 to 0.35	—	0.27 to 0.44	0.35 to 0.51	–0.51 to –0.35
**Self-awareness: mean 3.80 (0.56)**										
	*r*	0.28		0.56	0.64	0.37	0.36	1	0.41	–0.38
	95% CI	0.19 to 0.37		0.48 to 0.62	0.58 to 0.70	0.28 to 0.46	0.27 to 0.44	—	0.32 to 0.49	–0.46 to –0.29
**Self-efficacy: mean 4.25 (SD 0.52)**										
	*r*	0.26		0.46	0.44	0.37	0.43	0.41	1	–0.28
	95% CI	0.17 to 0.35		0.38 to 0.54	0.36 to 0.52	0.28 to 0.45	0.35 to 0.51	0.32 to 0.49	—	–0.37 to –0.18
**Stress: mean 2.94 (SD 0.66)**										
	*r*	–0.30		–0.51	–0.27	–0.26	–0.43	–0.38	–0.28	1
	95% CI	–0.39 to –0.21		–0.58 to –0.44	–0.36 to –0.18	–0.35 to –0.17	–0.51 to –0.35	–0.46 to –0.29	–0.37 to –0.18	—

^a^The CI is a plausible range of population correlations that could have caused the sample correlation [58]; all correlations are significant at *P*<.001.

^b^Not applicable.

**Table 3:** Outcome aggregated effects by assessment time point.^a^

Predictor	Sum of squares	Mean square	*df* (numerator)	*df* (denominator)	*F*	*P* value
Assessment time point	150.79	150.79	1	10149	459.40	<.001
Coaching outcome	482.45	60.31	8	10149	183.74	<.001
Assessment time point× coaching outcome	9.42	1.18	8	10149	3.59	<.001

^a^In this analysis, stress was reverse-coded to allow for a direct comparison by eliminating the chance our results would be overly skewed by stress being the one variable that decreases over time.

**Table 4:** Multilevel modeling regression by outcome.

Predictor			Emotional regulation		Life satisfaction		Prospection		Purpose and meaning		Resilience		Self-awareness		Self-efficacy		Social connection		Stress
Intercept (95% CI)			3.53 (3.46 to 3.61)		3.95 (3.89 to 4.02)		3.72 (3.65 to 3.78)		4.08 (4.01 to 4.15)		3.57 (3.50 to 3.64)		3.63 (3.57 to 3.70)		4.11 (4.06 to 4.17)		3.95 (3.90 to 4.00)		3.04 (2.96 to 3.11)
Assessment time point, β (95% CI)			.18 (.15 to .21)		.13 (.10 to .16)		.19 (.16 to .23)		.08 (.05 to .11)		.18 (.15 to .21)		.17 (.14 to .20)		.13 (.11 to .16)		.15 (.12 to .18)		–.10 (–.14 to .06)
**Random effects**																			
	σ2	0.22		0.16		0.20		0.18		0.18		0.14		0.13		0.14		0.30	
	τ00 (ID)	0.34		0.28		0.28		0.30		0.34		0.27		0.23		0.16		0.34	
	ICC^a^	0.60		0.64		0.59		0.62		0.65		0.66		0.64		0.54		0.53	
	Marginal R^2^	0.038		0.026		0.049		0.009		0.039		0.046		0.032		0.046		0.010	

^a^ICC: intraclass correlation coefficient.

### Longitudinal Growth Trajectories

The magnitude of growth over time and the growth ratios for each outcome are shown in Table 5. The growth ratios are ordered from the largest relative growth during the first time period (T1 to T2) relative to the second time period (T2 to T3) through to the largest relative growth during the second time period relative to that of the first. A growth ratio greater than 1.0 indicates that larger relative growth occurred between T1 and T2 than between T2 and T3 (ie, rapid initial growth), whereas a growth ratio less than 1.0 indicates that larger relative growth occurred between T2 and T3 (ie, delayed growth). A growth ratio of 1.0 indicates linear and sustained growth across both periods of time.

**Table 5:** Longitudinal growth ratios ordered by effect size.

Variable	Cohen *d*		Growth ratio
	T1–T2	T2–T3	
Prospection	0.43	0.13	3.41
Self-awareness	0.38	0.15	2.47
Self-efficacy	0.30	0.14	2.09
Social connection	0.30	0.24	1.27
Stress	0.13	0.11	1.24
Emotional regulation	0.27	0.22	1.22
Life satisfaction	0.20	0.20	1.01
Resilience	0.21	0.28	0.73
Purpose and meaning	0.08	0.16	0.48

**Figure 3:**
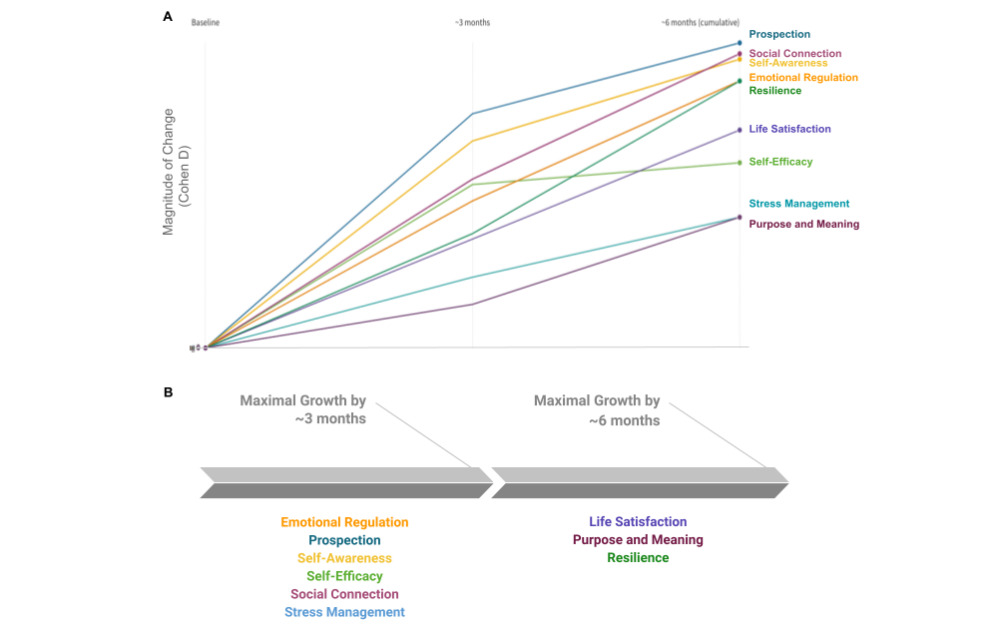
Growth magnitude throughout the intervention. (A) The effect size for each outcome from T1 to T2 and T2 to T3 is shown as the change from zero (T1) prior to the coaching intervention to approximately 3 months after beginning the intervention, and then the cumulative effect size change after approximately 6 months in the intervention. (B) Each outcome measure is separated into the time period, T1 to T2 at ~3 months or T2 to T3 at ~6 months, during which the outcome experienced the largest change.

To compare the relative growth of each dimension within each time period and over the cumulative coaching experience, Figure 3 visualizes the changes in two different ways. In this figure, stress is reverse-coded and denoted as “stress management” to enable a better comparison with the other outcomes. Additionally, each dimension is shown in a different color to clearly differentiate change patterns over time.

Figure 3A shows the cumulative effect sizes (ie, magnitude of growth) in each measured outcome between assessment time points, denoted as change at approximately 3 months and again at approximately 6 months, respectively. From this figure, it is evident that BetterUp users experienced the largest growth in prospection throughout the coaching intervention. Moreover, this growth was the largest during the first time period from T1 to T2 (measured at approximately 3-4 months after coaching began) and continued to grow, but by a smaller amount, during the second time period (approximately 6 months after coaching began). Conversely, the smallest and slowest growth, albeit significant, is seen for purpose and meaning. Finally, Figure 3 shows the relatively consistent growth in stress management, life satisfaction, emotional regulation, and social connection, although the magnitude of growth in each of these dimensions varies.

We also sought to identify the point at which each dimension experiences its maximal change due to the coaching intervention. This result allows a practitioner to understand the minimal amount of time to invest in coaching to experience the largest growth, on average, in a particular dimension of positive mental health and psychological well-being. As shown in Figure 3B, six of the nine measured dimensions showed the largest growth in approximately 3 months of BetterUp coaching, from T1 to T2: emotional regulation, prospection, self-awareness, self-efficacy, social connection, and stress management. Each of these six dimensions continues to increase in the subsequent 3 months; however, the magnitude of growth was lower in the second time period. The remaining three dimensions, resilience, life satisfaction, and feelings of purpose and meaning, showed continued or greater growth between about 3 and 6 months of BetterUp coaching (T2 to T3). This trend suggests that these dimensions benefit from greater investment of time and work to fully develop.

### Post-hoc Analyses

The results of post-hoc analyses are reported in Table 6. Briefly, we observed a significant decrease in stress between T1 and T2 and no significant change between T2 and T3. Conversely, the purpose and meaning dimension only significantly increased between T2 and T3, but not from T1 to T2. The remaining outcomes showed significant growth in both time periods; however, as shown in Figure 1 and Table 5, the magnitude of these changes varied by dimension.

**Table 6:** Results of post-hoc analyses.

Outcome		Mean difference (SE)^a^	*t* (*df*=782)	*P* value
**Emotional regulation**				
	T1–T2	–0.205 (0.034)	–6.056	<.001
	T2–T3	–0.158 (0.034)	–4.674	<.001
**Life satisfaction**				
	T1–T2	–0.134 (0.029)	–4.700	<.001
	T2–T3	–0.129 (0.029)	–4.521	<.001
**Prospection**				
	T1–T2	–0.305 (0.032)	–9.685	<.001
	T2–T3	–0.084 (0.032)	–2.671	.02
**Purpose and meaning**				
	T1–T2	–0.052 (0.030)	–1.705	.20
	T2–T3	–0.111 (0.030)	–3.634	.001
**Resilience**				
	T1–T2	–0.151 (0.030)	–4.985	<.001
	T2–T3	–0.203 (0.030)	–6.693	<.001
**Self-awareness**				
	T1–T2	–0.245 (0.026)	–9.340	<.001
	T2–T3	–0.096 (0.026)	–3.673	<.001
**Self-efficacy**				
	T1–T2	–0.179 (0.025)	–7.098	<.001
	T2–T3	–0.083 (0.025)	–3.263	.003
**Social connection**				
	T1–T2	–0.174 (0.027)	–6.511	<.001
	T2–T3	–0.124 (0.027)	–4.637	*<*.001
**Stress**				
	T1–T2	0.110 (0.039)	2.812	.01
	T2–T3	0.087 (0.039)	2.223	.07

^a^Paired contrasts of estimated marginal means controlling for between-individual variance using the latter group as the comparison group; therefore, a negative estimate indicates that the outcome at T1 is lower than the outcome at T2, or that the outcome at T2 is lower than that at T3.

Overall, our results demonstrate that there are different growth rates across a range of mental health and psychological well-being dimensions during a longitudinal coaching engagement. These results provide evidence of substantial growth in personal and professional outcomes throughout multiple months of one-on-one virtual coaching, and that the growth in these variables is dynamic as an individual develops various mindsets and behaviors through the BetterUp intervention.

## Discussion

### Main Findings

In this study, we demonstrated that BetterUp’s one-on-one virtual coaching improved positive mental health and psychological well-being in as short as 3 months, and drove continued benefits when users were engaged in professional coaching over longer periods of time. This is the first study to examine and establish the time-dependent and multiphase changes in specific dimensions of positive mental health and psychological well-being through an employer-sponsored coaching program.

This study used repeated assessments at baseline, part way through the coaching intervention, and after completing the intervention to identify a rich time course of personalized growth. These detailed trajectories can better inform coaches, coachees, and employers about the timing and magnitude of the mental health and well-being changes they can expect from a coaching intervention. In our examination of a range of factors that comprise positive mental health and psychological well-being (self-awareness, self-efficacy, emotional regulation, prospection, purpose and meaning, and social connection) and those that are important for managing mental health (stress, resilience, and life satisfaction), we demonstrated a complex interplay between these variables with improvements in each occurring at different rates during a longitudinal coaching engagement.

### Coaching to Mitigate Poor Mental Health and Bolster Positive Mental Health

Most notably, we found immediate significant reductions in stress within approximately 3-4 months of coaching (T1 to T2) that numerically continued to decrease over time, but without further significant improvements. Simultaneously, there was significant and linear growth in life satisfaction, and a slightly delayed, yet significant, increase in resilience throughout the full coaching program. Together, development of these three dimensions demonstrates the potential role of coaching to reduce the incidence of poor mental health, while supporting the development of resources to enable longitudinal mental health and well-being [32-34,59]. The different growth rates also suggest that individuals should first focus on reducing stress before expecting ongoing improvements in life satisfaction or resilience. Additional work should explore whether the immediate and significant reductions in stress are necessary to detect continued growth in the other measured outcomes, thereby delineating whether effective management of stress with the help of a coach acts as a gateway to further growth in other dimensions of mental health.

Given the personal, organizational, and societal costs of poor mental health, our results demonstrate the broad and far-reaching positive impacts of personalized professional leadership coaching. The results demonstrated that the investment of time and resources in an activity such as personalized professional coaching buffers individuals from the daily personal and work stressors that can erode mental health over time; allows them to build the skills to adapt, recover, and grow from challenging situations; as well as improves their outlook on their life situation. Although the coaching engagement was not directed specifically at improving mental health, the behaviors and mindsets developed are foundational for healthy mental functioning in all aspects of life.

### Longitudinal Growth in Positive Psychological Well-being Through Coaching

We found the largest growth from T1 (baseline) to T2, relative to that occurring from T2 to T3, in prospection abilities, self-awareness, and self-efficacy. Collectively, these three dimensions capture an individual’s ability to understand the factors that will influence their own development through the coaching experience. Prospection involves an individual using their past experiences and current goals to plan for the future, to include identifying potential obstacles [60-62]. The visioning and goal-setting that define prospection would explain the early and rapid growth in this dimension with ~3-4 months of coaching, as these activities are core to the coaching relationship and start early in the coaching engagement for building the motivation to work on the mindsets and behaviors that will aid goal attainment. Self-awareness measures the conscious attention to focus on the self, whereas self-efficacy captures the belief that an individual can achieve their goals. These dimensions complete a motivational framework for goal-setting and goal achievement. Together, the large early growth in these three dimensions suggests that coaches are guiding individuals in an introspective assessment of their needs and goals (self-awareness), developing goals and the plan for achieving them while identifying potential barriers (prospection), and building the beliefs that this vision is attainable (self-efficacy). Thus, significant early improvement in these three dimensions of psychological well-being are foundational elements of the intentional mindset and behavioral change needed to support positive mental health.

We found significant growth in the two remaining dimensions of psychological well-being, emotional regulation and social connection, throughout the intervention, but with slightly larger growth in the first 3-4 months (T1 to T2) relative to the change observed from approximately 3-6 months (T2 to T3). Emotional regulation, which aligns with the autonomy dimension of the Ryff model of psychological well-being [31], defines how well we internally regulate our emotions, thoughts, and behaviors. Importantly, such regulation promotes resilience [63], which is evident in our study given the greatest amount of growth experienced with a lengthier intervention of approximately 3-6 months of coaching. The link between emotional regulation and resilience should be further explored to understand whether improved emotional regulation is a necessary precursor for increased resilience. Additionally, social connection, which involves building and maintaining close and trusting relationships, is a behavior that experiences continual growth with the help of a coach as the coachee evolves and shifts how they may engage in interpersonal interactions. Importantly, social connection itself is strongly correlated with well-being and with dimensions that mitigate negative mental health such as stress and resilience [64,65], indicating that continued growth in this dimension through coaching supports positive mental health.

Finally, we found that the psychologically demanding processes of finding purpose and meaning, as well as building resilience took the longest time to fully mature. Specifically, the increase in these dimensions from T2 to T3 was approximately 50% and 30% greater than that from T1 to T2, respectively (Table 5 and Figure 3A).

In particular, finding purpose in life is a complex dimension that includes developing and holding beliefs about meaning of self, behaviors, pursuits, and others both in the past and present. We found that BetterUp users experienced significant growth in this dimension, but it took the entire length of the intervention, approximately 6 months, to experience maximal change. Notably, this study ended after the third assessment; however, future work should examine whether the development of this mindset, and others, continues to increase or plateaus.

Across the range of outcomes, we found specific groupings of dimensions that improved at similar rates. Such groupings suggest an underlying link in the psychological, cognitive, or affective processes that support dimensions that change at similar rates. Although we were not able to test this causal hypothesis directly, such a question could be pursued in future experimental studies with a prospective design that includes a matched control group.

### Professional Coaching to Boost Mental Health and Well-being

The patterns of growth identified in this study indicate that the model of one-on-one virtual coaching enables improvement in certain mindsets and behaviors before others, which act as building blocks to enable later growth in other constructs. This trend is most clearly seen in two different dimensions: (1) in the immediate and significant reduction in stress after approximately 90 days of engaging with a professional coach and the maintenance of this lower stress over the subsequent 3 months; and (2) the delayed growth in resilience, as well as purpose and meaning, which both significantly increased after approximately 6 months of coaching, highlighting the benefit of continued engagement with a coach to achieve growth in an area with demonstrated ties to mental health and well-being [66].

It is also notable that the measures that were used in this study were not clinical assessments; thus, they reflect an evaluation of psychological, cognitive, and affective states that may provide early indicators of growing maladaptive behaviors or, conversely, improvements that support mental flourishing that should be of interest to employers. However, in unpublished work (see Methods for more details), we identified a significant negative correlation between the PHQ-4, a brief screening tool for depression and anxiety, with resilience, life satisfaction, prospection, social connection, and meaning and purpose. This suggests that by improving these outcomes, there is an increased likelihood of a concomitant reduction in symptoms of depression and anxiety that may prevent a worsening of mental health. By capturing the effectiveness of longitudinal coaching to improve all of these outcomes, we suggest that an intervention such as one-on-one coaching can actually stop, or even reverse, the worsening of mental health. In particular, the significant reduction in stress and the continued increase in resilience while working with a professional coach focused on leadership development highlight that virtual one-on-one coaching can be used to mitigate a decline in mental health, while simultaneously building the skills to maintain or even improve mental health during challenging times, even when this is not the intended goal.

### Limitations and Future Work

Despite our finding of growth in various dimensions of mental health and well-being for BetterUp users, we recognize a few limitations of this study. Because of the retrospective and observational study design, we are unable to draw a causal link between the role of coaching on the different outcome measures. Subsequent research could include a noncoached control group that is assessed over the same period of time without any intervention and a noncoached control group that is provided self-guided materials intended to develop the same outcomes.

Our study design also required us to focus on individual outcomes separately, rather than looking at the temporal interplay between them. Such an analysis would have required a group that did not receive coaching, along with measures taken at higher frequency to enable a finer-grained time-series analysis with the appropriate level of statistical power. This type of design would also demand fewer outcome measures to maintain robust power while examining all interactions. Therefore, we consider this study to provide the first evidence of the specific outcomes that are more or less amenable to change through coaching and at what time point these changes would occur in the process. In particular, prospection, as well as purpose and meaning, showed the extremes of rapid and delayed growth, respectively. Given the literature demonstrating the impact of poor mental health on organizations, the shifts in stress and resilience through employer-provided coaching may have widespread impacts on workforce engagement and productivity. These outcomes may provide the best targets for follow-up studies.

The mixed linear effects modeling approach employed in this study allowed us to examine the variance attributed to the fixed effects relative to random effects. Given our ability to partial out the fixed effects, it should be noted that the effect of the assessment time points was relatively small, which suggests that a myriad of other factors explain the change in each of the dimensions of well-being and mental health. These factors likely stem from the nature of a typical one-on-one coaching journey, which is highly individualized to the needs and goals of the coachee. Additional research should explore the impact of individual psychological, cognitive, affective, or social factors; different rates of learning; or differential success in practicing coached dimensions in everyday life to further explain the mechanism by which one-on-one coaching is effective.

The decision to examine a cohort that was actively engaged in professional coaching through a virtual platform introduced two additional constraints: the use of self-report measures and a highly motivated population that is more likely to experience greater levels of growth. With respect to the latter point, we actually believe that this is a strength of the applied setting, as this enabled observing real-life growth, while still capturing the varying uptake and impact across participants. With respect to the former point, future studies could seek out populations that would allow for physical access, thus enabling the direct observation of behavioral changes. Additionally, by taking the study out of a very applied context, researchers could examine the effect of varying levels of motivation and the interactions between different outcome measures. Such study elements would allow for gaining a deeper understanding of how individuals experience coaching and when coaching is most efficacious.

### Conclusions

In summary, we found distinct temporal patterns in how professional coaching provided by BetterUp influences a change in various positive mental health and psychological well-being outcomes. This study provides a novel look at how behavioral change can evolve over time with support and scaffolding provided by a professional coach. To our knowledge, this is the first study to use multiple assessments to capture the change within individuals from before starting coaching to midway through their interaction and after approximately 6 months of being engaged in coaching. The multiple repeated assessments allowed us to analyze and understand a timeline of what happens throughout the longitudinal coaching journey.

The results reported herein can be used as a guide for coaches to stage the type of support they provide to their clients, which can enable early mental and behavioral changes that support improvement in skills that develop more slowly. Additionally, we identified professional coaching as a resource for employees that escapes the stigma associated with more traditional employee assistance and well-being programs. Professional one-on-one coaching empowers individuals to tackle a range of negative to positive mental health states with ongoing, personalized support that leaves them best prepared to excel personally and professionally, while contributing to the broader success of their organization.

## Appendix

Multimedia Appendix 1 Scale development technical report.

Multimedia Appendix 2 R script for reported analyses.

## Abbreviations

ANOVA: analysis of variance
PERMA: positive emotion, engagement, relationships, meaning, accomplishments
PHQ-4: Patient Health Questionnaire 4

## References

[1] Kortum E, Leka S, Cox T (2010). Psychosocial risks and work-related stress in developing countries: health impact, priorities, barriers and solutions. Int J Occup Med Environ Health.

[2] Peacock A, Leung J, Larney S, Colledge S, Hickman M, Rehm J, Giovino GA, West R, Hall W, Griffiths P, Ali R, Gowing L, Marsden J, Ferrari AJ, Grebely J, Farrell M, Degenhardt L (2018). Global statistics on alcohol, tobacco and illicit drug use: 2017 status report. Addiction.

[3] Steel Z, Marnane C, Iranpour C, Chey T, Jackson JW, Patel V, Silove D (2014). The global prevalence of common mental disorders: a systematic review and meta-analysis 1980-2013. Int J Epidemiol.

[4] Chisholm D, Sweeny K, Sheehan P, Rasmussen B, Smit F, Cuijpers P, Saxena S (2016). Scaling-up treatment of depression and anxiety: a global return on investment analysis. Lancet Psychiatry.

[5] Besteher B, Gaser C, Nenadić I (2020). Brain structure and subclinical symptoms: a dimensional perspective of psychopathology in the depression and anxiety spectrum. Neuropsychobiology.

[6] Cukrowicz KC, Schlegel EF, Smith PN, Jacobs MP, Van Orden KA, Paukert AL, Pettit JW, Joiner TE (2011). Suicide ideation among college students evidencing subclinical depression. J Am Coll Health.

[7] Fergusson DM, Horwood LJ, Ridder EM, Beautrais AL (2005). Subthreshold depression in adolescence and mental health outcomes in adulthood. Arch Gen Psychiatry.

[8] Gould CE, Karna R, Jordan J, Kawai M, Hirst R, Hantke N, Pirog S, Cotto I, Schussler-Fiorenza Rose SM, Beaudreau SA, O'Hara R (2018). Subjective but not objective sleep is associated with subsyndromal anxiety and depression in community-dwelling older adults. Am J Geriatr Psychiatry.

[9] Zhu Y, Qi S, Zhang B, He D, Teng Y, Hu J, Wei X (2019). Connectome-based biomarkers predict subclinical depression and identify abnormal brain connections with the lateral habenula and thalamus. Front Psychiatry.

[10] Friedman EM, Ryff CD (2012). Living well with medical comorbidities: a biopsychosocial perspective. J Gerontol B Psychol Sci Soc Sci.

[11] Keyes CLM, Dhingra SS, Simoes EJ (2010). Change in level of positive mental health as a predictor of future risk of mental illness. Am J Public Health.

[12] Montpetit MA, Bergeman CS, Bisconti TL, Rausch JR (2006). Adaptive change in self-concept and well-being during conjugal loss in later life. Int J Aging Hum Dev.

[13] Ryan RM, Deci EL (2001). On happiness and human potentials: a review of research on hedonic and eudaimonic well-being. Annu Rev Psychol.

[14] Sheldon KM, Lyubomirsky S (2006). Achieving sustainable gains in happiness: change your actions, not Your circumstances*. J Happiness Stud.

[15] Hone LC, Jarden A, Duncan S, Schofield GM (2015). Flourishing in New Zealand workers: associations with lifestyle behaviors, physical health, psychosocial, and work-related indicators. J Occup Environ Med.

[16] Boehm JK, Lyubomirsky S (2008). Does happiness promote career success?. J Career Assess.

[17] Attridge M, Gatchel RJ, Schultz LZ (2012). Employee assistance programsvidencecurrent trends. Handbook of Occupational Health and Wellness.

[18] Attridge M (2019). A global perspective on promoting workplace mental health and the role of employee assistance programs. Am J Health Promot.

[19] Attridge M, Cahill T, Granberry SW, Herlihy PA (2013). The national behavioral consortium industry profile of external EAP vendors. J Workplace Behav Health.

[20] Mattke S, Schnyer C, Van BK (2012). A review of the U.S. workplace wellness market. RAND Corporation.

[21] Pollitz K, Rae M (2016). Workplace Wellness Programs Characteristics and Requirements. Kaiser Family Foundation.

[22] Song Z, Baicker K (2019). Effect of a workplace wellness program on employee health and economic outcomes: a randomized clinical trial. JAMA.

[23] Eddington D, Pitts J (2015). Shared Values, Shared Results: Positive Organizational Health as a Win-Win Philosophy.

[24] Goetzel RZ, Henke RM, Tabrizi M, Pelletier KR, Loeppke R, Ballard DW, Grossmeier J, Anderson DR, Yach D, Kelly RK, McCalister T, Serxner S, Selecky C, Shallenberger LG, Fries JF, Baase C, Isaac F, Crighton KA, Wald P, Exum E, Shurney D, Metz RD (2014). Do workplace health promotion (wellness) programs work?. J Occup Environ Med.

[25] Joseph B, Walker A, Fuller-Tyszkiewicz M (2017). Evaluating the effectiveness of employee assistance programmes: a systematic review. Eur J Work Organiz Psychol.

[26] Kelloway EK (2017). Mental health in the workplace: Towards evidence-based practice. Can Psychol.

[27] Kirk AK, Brown DF (2006). Employee assistance programs: a review of the management of stress and wellbeing through workplace counselling and consulting. Austral Psychol.

[28] Jones RJ, Woods SA, Guillaume YRF (2015). The effectiveness of workplace coaching: A meta-analysis of learning and performance outcomes from coaching. J Occup Organ Psychol.

[29] Theeboom T, Beersma B, Van VA (2015). Coaching in Organizations? A Meta-Analytic Review of Individual Level Effects. Academy of Management Proceedings.

[30] Vanhove AJ, Herian MN, Perez ALU, Harms PD, Lester PB (2015). Can resilience be developed at work? A meta-analytic review of resilience-building programme effectiveness. J Occup Organ Psychol.

[31] Ryff CD (1989). Happiness is everything, or is it? Explorations on the meaning of psychological well-being. J Person Soc Psychol.

[32] Schneiderman N, Ironson G, Siegel SD (2005). Stress and health: psychological, behavioral, and biological determinants. Annu Rev Clin Psychol.

[33] Bonanno GA (2004). Loss, trauma, and human resilience: have we underestimated the human capacity to thrive after extremely aversive events?. Am Psychol.

[34] Diener E (1984). Subjective well-being. Psychol Bull.

[35] Ryff CD (2014). Psychological well-being revisited: advances in the science and practice of eudaimonia. Psychother Psychosom.

[36] Ellinger AD, Ellinger AE, Keller SB (2003). Supervisory coaching behavior, employee satisfaction, and warehouse employee performance: A dyadic perspective in the distribution industry. Hum Resource Dev Quart.

[37] Hamlin RG, Ellinger AD, Beattie RS (2006). Coaching at the heart of managerial effectiveness: A cross-cultural study of managerial behaviours. Hum Resource Dev Int.

[38] Ting S, Hart E (2004). Formal coaching. The Center for Creative Leadership handbook of leadership development.

[39] Salas E, Kozlowski S (2010). Learning, training, and development in organizations: Much progress and a peek over the horizon.

[40] Grant A (2001). Towards a psychology of coaching: the impact of coaching on metacognition, mental health and goal attainment.

[41] (2020). Core Competencies. International Coaching Federation.

[42] Ryff CD, Singer B (2016). Interpersonal flourishing: a positive health agenda for the new millennium. Pers Soc Psychol Rev.

[43] Ryff CD, Keyes CLM (1995). The structure of psychological well-being revisited. J Person Soc Psychol.

[44] Morozink JA, Friedman EM, Coe CL, Ryff CD (2010). Socioeconomic and psychosocial predictors of interleukin-6 in the MIDUS national sample. Health Psychol.

[45] Hutchinson D, Waters S, Yost A, Sinar E, Kellerman GR (2019). Whole Person Model 2.0 Technical Documentation.

[46] Avolio BJ, Gardner WL (2005). Authentic leadership development: getting to the root of positive forms of leadership. Leadership Quart.

[47] Luthans F, Luthans KW, Luthans BC (2004). Positive psychological capital: beyond human and social capital. Business Horizons.

[48] Seligman MEP (2011). Flourish: A visionary new understanding of happiness and well-being.

[49] Luszczynska A, Diehl M, Gutiérrez-Doña B, Kuusinen P, Schwarzer R (2004). Measuring one component of dispositional self-regulation: attention control in goal pursuit. Person Ind Diff.

[50] Diener E, Emmons RA, Larsen RJ, Griffin S (1985). The Satisfaction With Life Scale. J Pers Assess.

[51] Lips-Wiersma M, Wright S (2012). Measuring the meaning of meaningful work. Group Organiz Manag.

[52] Kroenke K, Spitzer RL, Williams JBW, Löwe B (2009). An ultra-brief screening scale for anxiety and depression: the PHQ-4. Psychosomatics.

[53] Löwe B, Wahl I, Rose M, Spitzer C, Glaesmer H, Wingenfeld K, Schneider A, Brähler E (2010). A 4-item measure of depression and anxiety: validation and standardization of the Patient Health Questionnaire-4 (PHQ-4) in the general population. J Affect Disord.

[54] Bates D, Mächler M, Bolker B, Walker S (2015). Fitting linear mixed-effects models using lme4. J Stat Soft.

[55] Gueorguieva R, Krystal JH (2004). Move over ANOVA: progress in analyzing repeated-measures data and its reflection in papers published in the Archives of General Psychiatry. Arch Gen Psychiatry.

[56] Kuznetsova A, Brockhoff PB, Christensen RHB (2017). LMER test package: tests in linear mixed effects models. J Stat Soft.

[57] Nakagawa S, Johnson PCD, Schielzeth H (2017). The coefficient of determination and intra-class correlation coefficient from generalized linear mixed-effects models revisited and expanded. J R Soc Interface.

[58] Cumming G (2014). The new statistics: why and how. Psychol Sci.

[59] Huppert F, Huppert FA, Baylis N, Keverne B (2005). Positive mental health in individuals and populations. The science of well-being.

[60] Gilbert DT, Wilson TD (2007). Prospection: experiencing the future. Science.

[61] Seligman MEP, Railton P, Baumeister RF, Sripada C (2013). Navigating into the future or driven by the past. Perspect Psychol Sci.

[62] Baumeister RF, Vohs KD, Oettingen G (2016). Pragmatic prospection: how and why people think about the future. Rev Gen Psychol.

[63] Tugade MM, Fredrickson BL (2006). Regulation of positive emotions: emotion regulation strategies that promote resilience. J Happiness Stud.

[64] Bliese PD, Edwards JR, Sonnentag S (2017). Stress and well-being at work: a century of empirical trends reflecting theoretical and societal influences. J Appl Psychol.

[65] House J (1981). The nature of social support. Work stress and social support.

[66] Steger MF, Dik BJ, Duffy RD (2012). Measuring meaningful work. J Career Assess.

