# Supplementary material for: Time to Change for Mental Health and Well-being via Virtual Professional Coaching: Longitudinal Observational Study
Source: J Med Internet Res. 2021 Jul 5;23(7):e27774. doi: 10.2196/27774 (PMC8406100; doi:10.2196/27774)
Supplement: Multimedia Appendix 1 [file jmir_v23i7e27774_app1.pdf]

BetterUp

1200 Folsom St, San Francisco, CA 94103

Scale Development Technical Documentation

Table of Contents

Background....................4
Vision for BetterUp Assessments....................4
Review of the Literature....................5
Method....................7
Sample....................7
Scale Development....................7
Analytic Approach Overview....................8
Model Development and Validation....................10
Phase 1: Item Analysis....................10
Phase 2: Final Dimension Correlation....................10
Phase 3: Dimension Discriminant and Convergent Validity....................10
Phase 4: Demographic Comparisons....................11
Gender....................11
Ethnicity....................11
Phase 5: Test-Retest....................12
One-Month Retest....................12
Three-Month Retest....................12
Phase 6: Science Board Review....................12
Summary of Key Findings....................13
Appendix A: Tables....................14
Table 1: Sample Demographics....................15
Table 2: Categorical Demographic Variables....................16
Table 4: Intercorrelations of Dimensions....................18
Table 5: Divergent & Discriminant Validity....................19
Table 6: Descriptives and Univariate t-Tests of Dimensions by Gender....................20
Table 7: Descriptives of Dimensions by Ethnicity....................21
Appendix B: Scale Definitions....................22
Operational Definitions....................23

© BetterUp Inc. 2019

Background

Vision for BetterUp Assessments

BetterUp's mission is to help every professional lead their life with greater clarity, purpose, and passion. Self-exploration and self-discovery are powerful components of the coaching process. Coaches often employ a range of tools to support client self-exploration. These tools vary in the degree of structure and facilitation required. Common tools include self-observation exercises, journaling, behavioral practices, topic-specific exercises, mindfulness exercises, and topic-specific educational tools (e.g., readings, videos).

Another broad category of coaching tools is psychometric assessments. Assessments are a common tool used to facilitate self-discovery. They share many of the features of less structured/formal tools mentioned above; however, they typically have the added benefit of being grounded in theory and having measurable properties of quality such as reliability and validity.

If the end goal is facilitating self-discovery and awareness, and ultimately transforming behavior, assessments need to be accurate to be valuable tools in helping our coachees (referred to as BetterUp members) take action. Sound psychometric assessments can provide valuable information to coaches to inform their approach. Thus, the reliability and validity of these assessments are critical. In addition to facilitating the coaching process, accurate and reliable assessments help organizations (referred to as BetterUp partners) evaluate the impact of coaching at both an individual and aggregate levels by measuring growth throughout the engagement.

Psychometric assessments can be used to measure a broad variety of individual differences. Examples of common assessment types include personality, interests, preferences, skills, performance, knowledge, and abilities. By capitalizing upon an assessment's ability to provide different lenses through which a client can view him or herself – in combination with the support of a coach – we can build a personalized self-discovery experience for our coachees. The suite of assessments described in this technical report will create a psychometrically sound assessment library that can be leveraged by organizations, coaches, and coachees to both facilitate and track growth.

While the assessment library will be built with flexibility and personalization in mind, we want to do so in a way that does not impede obtaining a holistic perspective or limit BetterUp's ability to accumulate knowledge. To balance these priorities, coachees will receive a common set of assessments at certain points in their coaching experience (e.g., at onboarding and at predetermined "reflection points"), referred to as "core" assessments. Additional assessment content from the library can then be delivered to personalize the coachees' experience through push (e.g., assigned by the coach, automatically suggested based on where they are in their development journey, or assigned by their organization) or pull (coachee initiated/selected) mechanisms. Ultimately, the ability to consistently provide new personal insights is expected to

© BetterUp Inc. 2019

(a) enhance coachee engagement, (b) serve as a coaching tool to help tailor coaching and, (c) through use of diverse, personalized, and engaging assessments provide additional data to feed ongoing development. In sum, these changes should create a more integrated experience across coaching and other platform components, such as assessments, extended network (i.e., specialist coaching), and smart learning.

Review of the Literature

We developed the guiding framework of WPM 2.0 based on a thorough review of research published in top-tier academic journals. During the literature review, we focused on recent, cutting edge advancements. Given BetterUp's mission to help professionals lead their lives with greater clarity, purpose, and passion, we specifically focused on research on goal-setting, well-being, and leadership. These literatures informed the outcomes we selected to measure in the model.

The core outcomes we identified are outlined below:

Self-awareness. Self-awareness is the extent to which we direct our consciousness to focus on the self.[1] Self-awareness relates to the goal component of Motivational Systems Theory. We must be aware of our behaviors in order to set strategic personal goals and work towards achieving them.[2] BetterUp's data suggest that self-awareness is one of the most foundational topics discussed in coaching sessions, and research suggests that self-awareness coupled with goal-setting is predictive of high performance.[3]

Self-efficacy. Self-efficacy refers to individuals' beliefs about their capabilities to achieve their goals.[4] In Motivational Systems Theory, self-efficacy is a personal agency belief that helps predict and explain individuals' behaviors. Research suggests that self-efficacy is associated with stronger effort, persistence, and goal attainment.[5]

Social Connection. The extent to which we remain close and engaged with important, supportive people in our lives. By maintaining our close personal relationships, we feel a heightened sense of belonging, and we receive social support that enhances our well-being.[6]

Prospection. The extent to which we pragmatically think through ways to achieve our future goals. By envisioning desired future states and figuring out how to turn those desires into reality, we are able to enhance personal meaning and motivation.[7]

---

[1] Duval, S., & Wicklund, R. A. (1972). A theory of objective self awareness.
[2] Ridley, D. S., Schutz, P. A., Glanz, R. S., & Weinstein, C. E. (1992). Self-regulated learning: The interactive influence of metacognitive awareness and goal-setting. *The journal of experimental education*, *60*(4), 293-306.
[3] Ridley, D. S., Schutz, P. A., Glanz, R. S., & Weinstein, C. E. (1992). Self-regulated learning: The interactive influence of metacognitive awareness and goal-setting. *The journal of experimental education*, *60*(4), 293-306.
[4] Bandura, A. (1991). Social cognitive theory of self-regulation. Organizational behavior and human decision processes, 50(2), 248-287
5 Judge, T. A. (2009). Core self-evaluations and work success. Current Directions in Psychological Science, 18(1), 58-62. ; Zimmerman, B. J., Bandura, A., & Martinez-Pons, M. (1992). Self-motivation for academic attainment: The role of self-efficacy beliefs and personal goal setting. American educational research journal, 29(3), 663-676.
[6] House, J. S. (1981). The nature of social support. *Work Stress and Social Support*, 13-30.
[7] Baumeister, R. F., Vohs, K. D., & Oettingen, G. (2016). Pragmatic prospection: How and why people think about the future. Review of General Psychology, 20(1), 3-16.; See also Arbit (2018). Coaching for Pragmatic Prospection. BetterUp.

© BetterUp Inc. 2019

Emotional Regulation. The extent to which we regulate our emotions to remain calm and collected. The more emotionally stable we are, the better equipped we are to remain resilient and excel when challenges arise.[8]

Resilience. The extent to which individuals positively adapt in the context of negative or stressful experiences.[9] Resilience in the workplace is becoming increasingly important as novel demands such as new technologies and changing governmental policies become more prevalent.[10] In addition to its link with performance, resilience is associated with higher well-being.[11]

Stress. The extent to which individuals experience tension as a result of work and non-work demands. Work stress imposes an estimated $187 billion financial burden in the United States.[12] In addition to its detrimental toll on employee well-being, employee stress is associated with organizational health care costs and productivity losses.[13]

Purpose and Meaning. The extent to which individuals experience a sense of personal meaning to what they do at work. As mentioned earlier in the literature review, BetterUp research demonstrates that employees highly value meaningful work; on average, employees would take a substantial pay cut in exchange for more meaningful work.[14] Employers may also indirectly benefit from employee meaning and purpose through reduced turnover and increased performance.[15]

Life Satisfaction. The extent to which individuals make positive global assessments of the quality of their lives.[16] Life satisfaction is a cognitive, judgmental process that centers on individuals' own evaluations. Because life satisfaction is a subjective experience, it can be influenced by one's mindsets and behaviors without changes in the external environment[17]

---

[8] Tugade, M. M., & Fredrickson, B. L. (2007). Regulation of positive emotions: Emotion regulation strategies that promote resilience. Journal of happiness studies, 8(3), 311-333.
[9] Luthar, S. S., Cicchetti, D., & Becker, B. (2000). The construct of resilience: A critical evaluation and guidelines for future work. Child development, 71(3), 543-562.
[10] Oishi, S., & Diener, E. (2009). Goals, culture, and subjective well-being. In *Culture and well-being* (pp. 93-108). Springer, Dordrecht.
[11] Oishi, S., & Diener, E. (2009). Goals, culture, and subjective well-being. In *Culture and well-being* (pp. 93-108). Springer, Dordrecht.
[12] Hassard, J., Teoh, K. R. H., Visockaite, G., Dewe, P., & Cox, T. (2018). The cost of work-related stress to society: A systematic review. *Journal of Occupational Health Psychology*, *23*(1), 1–17.
[13] Hassard, J., Teoh, K. R. H., Visockaite, G., Dewe, P., & Cox, T. (2018). The cost of work-related stress to society: A systematic review. *Journal of Occupational Health Psychology*, *23*(1), 1–17.
[14] https://www.betterup.co/en-us/about/news-press/press-releases/workers-value-meaning-at-work-new-research-from-betterup-shows-just-how-much-theyre-willing-to-pay-for-it/
[15] https://www.betterup.co/en-us/about/news-press/press-releases/workers-value-meaning-at-work-new-research-from-betterup-shows-just-how-much-theyre-willing-to-pay-for-it/
[16] Diener, E. D., Emmons, R. A., Larsen, R. J., & Griffin, S. (1985). The satisfaction with life scale. *Journal of personality assessment*, *49*(1), 71-75.
[17] Diener, E. D., Emmons, R. A., Larsen, R. J., & Griffin, S. (1985). The satisfaction with life scale. *Journal of personality assessment*, *49*(1), 71-75.

© BetterUp Inc. 2019

Method

Sample

The validation effort recruited 1030 qualified MTurk workers for participation. MTurk is a platform that provides relatively quick online access to large samples of workers.[18] Research suggests that MTurk workers are generally representative of the U.S. population. MTurk samples tend to be older, more ethnically diverse, and have more work experience than traditional college student samples.[19] Research has found that MTurk samples produce test-retest reliabilities and coefficient alphas similar to those of other research samples.[20]

We adopted the following methodology to ensure the MTurk sample is representative of the average BetterUp member. First, we administrated a short demographic survey and restricted invitations to participate in the study to individuals who work full-time on a team of three or more individuals, for whom English is a primary language, and who have MTurk worker approval scores above 95%.[21] Additionally, all participants eligible for inclusion in this validation study had previously completed other studies with BetterUp. This gave us access to additional assessments beyond what was initially included in the WPM 2.0 validation efforts. Overall, our sample was 57% male, an average of 39 years of age, had an average job tenure of 6 years, and worked an average of 42 hours per week. For a full demographic and industry breakdown of sample participants refer to Tables 1 - 3.

Scale Development

To develop the scales, members of BetterUp Labs followed best practices as outlined in *The Standards for Educational and Psychological Testing*[22], which describe four sources of validity evidence: (a) test content, (b) response processes, (c) internal structure, and (d) relations to other measures.

The first step in designing the scales was to identify constructs to include in the model. Using the knowledge gained from the validation of earlier scales, we conducted an updated review of the literature and discussions with key stakeholders,[23] and a hypothesized model was developed. A team of nine industrial organizational psychologists including BetterUp Labs team members, contractors, and university faculty contributed to this effort.

---

[18] Buhrmester, M. D., Talaifar, S., & Gosling, S. D. (2018). An evaluation of Amazon's Mechanical Turk, its rapid rise, and its effective use. *Perspectives on Psychological Science*, *13*(2), 149-154.
[19] Behrend, T. S., Sharek, D. J., Meade, A. W., & Wiebe, E. N. (2011). The viability of crowdsourcing for survey research. *Behavior research methods*, *43*(3), 800.
[20] Buhrmester, M., Kwang, T., & Gosling, S. D. (2011). Amazon's Mechanical Turk: A new source of inexpensive, yet high-quality, data?. *Perspectives on psychological science*, *6*(1), 3-5.
[21] Mturk approval score is the percentage of assignments submitted that have been approved.
[22] American Educational Research Association, American Psychological Association, National Council on Measurement in Education, Joint Committee on Standards for Educational, & Psychological Testing (US). (2014). *Standards for educational and psychological testing.* Washington, DC: American Educational Research Association.
[23] Perspectives of key stakeholders obtained by administering a survey to 8 partners, 39 coaches, and 23 BetterUp employees regarding the competencies and behavioral states that are seen as most important, valuable, and relevant.

© BetterUp Inc. 2019

BetterUp psychologists then wrote operational definitions for all constructs identified. These definitions were developed through an extensive review of the literature. Items were then written to measure the relevant outcomes.[24] All items were compiled, reviewed, and revised by two separate team members. A post-review discussion was then held to resolve issues and remove items as needed. Because most of these scales were developed for this project, we identified existing scales previously validated in the literature to evaluate discriminant and convergent validity.

All Items included in this validation effort used one of these four different response scales:

Agreement. Agreement [1, 'Strongly Disagree'; 2, 'Disagree'; 3, 'Neither Disagree nor Agree'; 4, 'Agree'; 5, 'Strongly Agree']

Frequency. Frequency [1, 'Never'; 2, 'Rarely'; 3, 'Sometimes'; 4, 'Often'; 5, 'Very Often']

Extent. Extent [1, 'Not at all'; 2, 'Slightly'; 3, 'Moderately'; 4, 'Quite a bit'; 5, 'Extremely']

We chose to employ a variety of rating scales when creating our initial item pool to increase our ability to assess constructs from a variety of perspectives. Using a variety of response options also helped keep the assessment short by minimizing item redundancy and increased the likelihood that items would assess distinct portions of their intended construct.

Analytic Approach Overview

The primary goals in the development were to assure strong psychometric properties of the scales, to make the collection of scales both comprehensive and flexible, and to create a clear narrative for members and coaches.

Several phases were conducted to achieve these goals:

- Phase 1: Item Analysis
- Phase 2: Final Dimension Correlations
- Phase 3: Dimension Correlations with Marker Scales
- Phase 4: Demographic Comparisons
- Phase 5: Test-Retest
- Phase 6: Science Board Review

Phase 1 was intended to reduce the number of items through item analysis. Phase 2 assessed sub-dimension inter-correlations. Phase 3 assessed whether our newly developed sub-dimension

[24] Items were developed by drawing from WPM 1.0 items (where relevant), existing measures (where permission could be obtained), as well as custom item development by the BetterUp WPM 2.0 team. A final set of 345 items was created and administered to participants.

© BetterUp Inc. 2019

measures demonstrated the expected relationships with established measures of similarly validated constructs. Phase 4 assessed if there were differences in scores across demographic groups. Phase 5 assessed if scores on the assessments remained consistent 1 and 3 months after the initial survey. Finally, during phase 6 we sought feedback from the prominent scholars on BetterUp's science board to provide a final review of our validation efforts.

© BetterUp Inc. 2019

Model Development and Validation

Phase 1: Item Analysis

The primary goal of this phase of the validation was to reduce the number of items per subscale by removing poorly performing items based on various classical test theory (CTT) item statistics. This was determined by evaluating means, standard deviations, skewness, kurtosis, inter-item correlations, item-total correlations, and Cronbach's alpha. Items were considered poorly performing if their means were too high, standard deviations were too low, skewness and kurtosis values were too high, or inter-item / item-total correlations were too low (in both relative and absolute terms). Overall, the purpose of evaluating these statistics was to test whether the items within each scale correlated highly enough such that a total-score was appropriate for each item set. Additionally, these analyses allowed us to reduce the number of items administered. The final operational subscale definitions and their reliabilities can be found in Appendix B.

Through CTT we were able to trim each scale down from 3-10 items to 2-4 items. All multi-item scales retained an acceptable internal consistency reliability.

Phase 2: Final Dimension Correlation

We next evaluated the correlations between each of our newly developed measures (Table 4). As expected, the vast majority of our dimensions were related.

Phase 3: Dimension Discriminant and Convergent Validity

In addition to examining the patterns of correlations between dimensions, we also examined the relationships between the selected dimensions and measures previously validated in the well-being literature (i.e., marker scales). This included the Authentic Leadership Questionnaire (ALQ)[25], Psychological Capital (PsyCap)[26], and Seligman's PERMA[27].

Table 5 present the correlations between each dimension and the marker variables included in this validation study.

Overall, these analyses provided further evidence that the new assessments accurately and comprehensively assess the well-being construct space.

[25] Avolio, Bruce J., and William L. Gardner. "Authentic leadership development: Getting to the root of positive forms of leadership." The leadership quarterly 16.3 (2005): 315-338.
[26] Luthans, F., Luthans, K. W., & Luthans, B. C. (2004). Positive psychological capital: Beyond human and social capital.
[27] Seligman, M. E., & Flourish, P. (2011). A visionary new understanding of happiness and well-being. *New York*.

© BetterUp Inc. 2019

BetterUp

Phase 4: Demographic Comparisons

Several multivariate analyses of variance (MANOVAs) were conducted to detect differences across gender and ethnicity on the different dimensions. MANOVAs were chosen as a first step due to the moderate amounts of multicollinearity among the dimensions and as an attempt to control the family-wise error rate. Using a MANOVA provides an indication of the magnitude of differences across the components between these demographic groups. In the following analyses, we interpret the multivariate test statistic, Wilks Lamba (Λ), and the effect size indicator, partial eta squared (η_p^2). Established cutoffs suggest that a η_p^2 value greater than or equal to .01 can be considered a small effect size, .06 can be considered a medium effect size, and an effect size greater than .14 can be considered large. [28]

All variables demonstrated linearity and acceptable univariate normality. However, all Box's M tests were significant which suggests there exists inequality of the covariance matrices across groups. When given sufficiently large sample sizes, MANOVAs are robust to this violation; however, in the ethnicity analyses there is a large degree of unevenness in the sample sizes across groups. In these cases, this assumption violation can result in significance testing that is either too liberal or too conservative. As a follow-up to a significant MANOVA, we computed *t*-tests for demographic variables consisting of two groups (i.e., gender), and performed an appropriate post-hoc test for the ethnicity variable that included four groups.

Gender

The multivariate analysis found there was significant differences attributed to gender $\Lambda = .86$, $F(25, 1003) = 6.62$, $p < .001$, $\eta_p^2 = 0.14$. This meets the criterion for a large effect size. Results suggested that approximately 14% of the variance of the dimensions can be explained by gender.

Univariate *t*-tests were performed to explore differences between men and women across the dimensions (Table 6). Women rated themselves significantly higher across several beneficial WPM 2.0 components and lower on several harmful components. The largest differences were in Emotional Regulation, Stress, and Resilience.

Ethnicity

The multivariate analyses found statistically significant differences attributed to ethnicity $\Lambda = .90$, $F(75, 2903) = 1.31$ $p < .05$, $\eta_p^2 = 0.03$). Although significant, these results suggested that only 3% of the variance was explained by participants' ethnicity. This is the smallest amount of variance explained by the demographic variables assessed. Means and standard deviation for each group across all dimensions can be found in Table 7.

Given the unequal variances and sample sizes discussed previously, a Games-Howell post hoc test was performed to investigate group differences. The largest of the statistically significant

[28] Cohen J. (1988). Statistical Power Analysis for the Behavioral Sciences. New York, NY: Routledge Academic

© BetterUp Inc. 2019

differences included: Participants identifying as Black reported higher levels Prospection, and participants identifying as Asian reported the most self-efficacy. Overall, all of the differences were small in magnitude.

Phase 5: Test-Retest

The next step in the validation process was to complete two separate test-retest reliability studies. The first was completed one month and the second was completed three months after the original assessment. Collecting test-retest data allowed us to measure changes in scores in an uncoached sample and evaluate the stability of these metrics. In the absence of coaching, we expect our assessments to demonstrate minimal change over time.

One-Month Retest

We first examined test-retest correlations of each of the dimensions. The test-retest correlations were moderate to high (rs = .64-.85), as expected. We then examined individuals' change scores on each dimension across time. Paired sample t-tests demonstrated that the average change scores were not significantly different from zero. The high degree of stability in these scales over time and the minimal change in the absence of coaching is a critical finding for evaluating change scores among BetterUp members going through coaching, where we would expect to see changes in scores over time.

Three-Month Retest

The test-retest correlations from Time 1 (original assessment) to Time 3 (three months later) were moderate to high (rs = .63-.90), as expected. Just as before, we then examined individuals' change scores on each dimension across time. Paired sample t-tests demonstrated that the average change scores were significantly different from zero for prospection. However, the effect sizes of the significant differences was small (d = .11). Together, the findings support our hypotheses that our measures are reliable over a three-month period. It's not surprising that the measures were not as highly correlated across a three-month span compared to the one-month comparison, yet they still demonstrated a high degree of stability.

Phase 6: Science Board Review

After completing the previous phases of the validation efforts, BetterUp's Science Board conducted a final review of our validation efforts. BetterUp's Science Board includes luminaries from across scientific disciplines including psychology, business, and management. The Science Board was created to ensure that BetterUp benefit from broad insight into core scientific, business, and technological trends, and helps shape our short and long-term business strategies. As another layer expert input, the Science Board was asked to review the technical approach and the assessments. This was intended to provide an additional independent review by highly respected scientists who were not involved in the validation work.

© BetterUp Inc. 2019

Overall, the assessments were received positively by our Science Board members. Feedback mainly centered on the nomenclature of the individual psychological constructs of the assessment, clarity of specific items, and considerations for future content and research. Feedback was reviewed by the validation team and implemented where possible or noted for future research. The Science Board review process helped ensure that our validation efforts reached the highest standards for psychometric testing and scientific excellence.

Summary of Key Findings

Our validation efforts supported reduced model redundancies and length, validated our newly developed measures against previously established assessments, and found few unexpected differences across demographic groups.

We assessed the relationships among all of the components and previously validated measures assessing similar psychological constructs. This included established leadership (ALQ), well-being (PERMA & PsyCap), and life satisfaction metrics. Overall, these analyses provide evidence that the collection of assessments are measuring what they were intended to measure.

Minor differences between demographic groups were found on the developed components. The majority of the differences were expected. The largest group differences were for gender. In our analyses of gender, we found that women self-rated as higher on Emotional Regulation, Resilience, and lower on Stress.

The test-retest analyses demonstrated that the measures are generally reliable over time. Test-retest correlations after one month ranged from .64 to .85. Test-retest correlations after three months ranged from .63 to .90. Paired sample *t*-tests suggested that the average change scores on the measures were not generally significantly different from zero across both one and three months, which is an important finding given that these participants did not receive coaching through BetterUp's platform.

Overall, the validation efforts of these dimensions, and subsequently supported through analyses of the benchmarking datasets, resulted in a comprehensive assessment comprising mindsets and behaviors that promote well-being, leadership behaviors, and the key outcomes. This will allow BetterUp to accurately provide feedback to members, coaches, and partners, and ultimately work to direct coaching in a way that is the most meaningful and developmentally impactful across all domains of life.

© BetterUp Inc. 2019

Appendix A: Tables

© BetterUp Inc. 2019

Table 1: Sample Demographics

Characteristic	M	SD
Age (years)	38.67	10.03
Job Tenure (years)	6.04	5.87
Hours Worked (weekly)	41.76	4.52
Salary	$64,319	$32,026

© BetterUp Inc. 2019

Table 2: Categorical Demographic Variables

Characteristic		n	%
Gender			
	Male	587	57
	Female	442	43
	Other	1	0
Ethnicity			
	Caucasian or White	809	78.5
	Asian	77	7.5
	Black or African American	57	5.5
	Latino or Hispanic	56	5.5
	Mixed or Other	26	2.5
	American Indian or Alaskan Native	2	.2
	Prefer not to Answer	3	.3
Manager status			
	Individual Contributor	545	53
	People Leader	485	47
Education			
	HS/GED Some college	207	20.1
	Associate Degree	109	10.6
	Bachelor's degree	487	47.3
	Master's degree	184	17.9
	Adv. Graduate work or Ph.D.	43	4.1
Household Income			
	$25,000 to $44,999	395	38.3
	$45,000 to $74,999	416	40.7
	$75,000 to $100,000	136	13.2
	more than $100,000	80	7.8
Industry			
	For-Profit	740	72
	Government	167	16.2
	Non-Profit	121	11.8
Organization Size (employees)			
	< 50	118	16.8
	50 – 100	105	11.3
	100 – 249	107	11.5
	250 – 499	132	14.2
	500 - 1,000	101	10.9
	1,000 - 4,999	94	10.1
	5,00 - 9,999	106	11.4
	> 10,000	167	18

Note: data for organizational size *n* = 930

© BetterUp Inc. 2019

Table 3: Participants by Industry

Industry	Specific Work Role	n	%
Business			
	Business and Financial Operations	124	12.1
	Management	74	7.2
	Marketing and Advertising	31	3
	Corporate Consulting	6	.6
Technology			
	Information Technology	117	11.4
	Software Engineering	31	3
Healthcare			
	Healthcare Support	52	5.1
	Healthcare Practitioner and Technical	44	4.3
	Education, Training, and Library	112	10.9
Math, Science, and Education	Life, Physical, and Social Science	33	3.2
	Applied Math and Statistical Analysis	9	.9
	Production	29	2.8
	Transportation and Material Moving	29	2.8
Construction	Architecture and Engineering	21	2
	Construction and Extraction	11	1.1
	Installation, Maintenance, and Repair	11	1.1
	Sales and Related	84	8.2
	Office and Administrative Support	80	7.8
	Arts, Design, Entertainment, and Media	36	3.5
	Food Preparation and Serving	24	2.3
Other Industry	Community and Social Services	23	2.2
	Legal	22	2.1
	Protective Service	11	1.1
	Personal Care and Service	5	.5
	Building and Grounds Cleaning	5	.5
	Farming, Fishing, and Forestry	4	.4

© BetterUp Inc. 2019

Table 4: Intercorrelations of Dimensions

	1	2	3	4	5	6	7	8	9
1. Social Connection	1								
2. Emotional Regulation	0.358	1							
3. Prospection	0.510	0.405	1						
4. Stress	-0.354	-0.464	-0.291	1					
5. Purpose & Meaning	0.397	0.344	0.372	-0.282	1				
6. Life Satisfaction	0.560	0.478	0.497	-0.539	0.560	1			
7. Resilience	0.364	0.639	0.447	-0.517	0.414	0.613	1		
8. Self-Efficacy	0.489	0.524	0.563	-0.404	0.452	0.593	0.596	1	
9. Self-Awareness	0.417	0.434	0.566	-0.417	0.350	0.514	0.464	0.584	1

N=1030, all correlations significant at $p<.001$

© BetterUp Inc. 2019

BetterUp

Table 5: Divergent & Discriminant Validity

	ALQ				PsyCap					PERMA							Affect		SRS	LS
	Self-Awareness	Moral Perspective	Processing	Relational Transparent	Efficacy	Hope	Resilience	Optimism	Overall	Positive Emotion	Relation-ships	Meaning	Accomplish-ment	Negative Emotion	Health	Overall	Positive	Negative	Self-Regulation	Life Satisfaction
Social Connection	0.493	0.396	0.382	0.537	0.384	0.502	0.381	0.523	0.531	0.576	0.637	0.573	0.490	-0.370	0.352	0.627	0.474	-0.302		
Emotional Regulation	0.337	0.324	0.284	0.295	0.464	0.499	0.506	0.492	0.579	0.490	0.436	0.491	0.513	-0.534	0.372	0.518	0.429	-0.492		
Prospection	0.462	0.432	0.371	0.391	0.482	0.601	0.508	0.544	0.636	0.495	0.459	0.561	0.590	-0.334	0.341	0.583	0.531	-0.270		
Stress	-0.257	-0.241	-0.210	-0.294	-0.331	-0.419	-0.345	-0.477	-0.463	-0.599	-0.521	-0.524	-0.494	0.716	-0.463	-0.580	-0.339	0.562	-0.410	-0.460
Purpose & Meaning	0.391	0.372	0.328	0.356	0.387	0.499	0.357	0.513	0.523	0.483	0.447	0.578	0.496	-0.313	0.279	0.554	0.406	-0.238	0.380	0.410
Life Satisfaction	0.438	0.419	0.345	0.430	0.452	0.585	0.438	0.631	0.625	0.796	0.739	0.832	0.715	-0.579	0.518	0.847	0.522	-0.436		
Resilience	0.390	0.376	0.254	0.355	0.496	0.545	0.565	0.575	0.641	0.619	0.508	0.608	0.631	-0.573	0.442	0.647	0.495	-0.459	0.590	0.490
Self-Efficacy	0.497	0.453	0.326	0.412	0.532	0.607	0.519	0.570	0.664	0.603	0.531	0.629	0.637	-0.460	0.426	0.661	0.586	-0.410	0.610	0.510
Self-Awareness	0.444	0.438	0.301	0.384	0.427	0.518	0.463	0.427	0.547	0.492	0.503	0.578	0.565	-0.475	0.366	0.583	0.505	-0.377	0.580	0.370

N=1030, all correlations significant at p<.001

© BetterUp Inc. 2019

Table 6: Descriptives and Univariate t-Tests of Dimensions by Gender

	Men		Women				95% Confidence Interval		
	M	SD	M	SD	*t*-test	Δ M	Lower Bound	Upper Bound	Cohen's d
Social Connection	3.82	0.81	3.92	0.79	-1.92	-0.10	-0.20	0.00	-0.12
Emotional Regulation	3.93	0.84	3.59	0.95	6.03*	0.34	0.23	0.45	0.38
Strategic Planning	4.10	0.76	4.11	0.74	-0.23	-0.01	-0.10	0.08	-0.01
Stress	2.36	0.94	2.66	1.00	-5.04*	-0.31	-0.43	-0.19	-0.31
Purpose	3.85	1.01	3.94	1.02	-1.36	-0.09	-0.21	0.04	-0.09
Life Satisfaction	3.72	0.94	3.64	0.91	1.28	0.07	-0.04	0.19	0.09
Resilience	3.92	0.81	3.74	0.95	3.28*	0.18	0.07	0.29	0.20
Self-Efficacy	4.13	0.76	4.12	0.74	0.39	0.02	-0.07	0.11	0.01
Self-Awareness	4.05	0.68	4.02	0.69	0.67	0.03	-0.06	0.11	0.04

Note: Men n = 587; Women = 442; * $p < .05$

© BetterUp Inc. 2019

Table 7: Descriptives of Dimensions by Ethnicity

	Ethnicity	M	SD	Dimension	Ethnicity	M	SD
Emotional Regulation	Caucasian / White	3.76	0.91	Self-Efficacy	Caucasian / White	4.10	0.74
	Asian	3.68	0.92		Asian	4.04	0.76
	Black / African American	4.08	0.80		Black / African American	4.42	0.82
	Latino / Hispanic	3.88	0.90		Latino / Hispanic	4.26	0.73
Life Satisfaction	Caucasian / White	3.69	0.92	Self-Awareness	Caucasian / White	4.03	0.69
	Asian	3.53	0.92		Asian	3.91	0.70
	Black / African American	3.83	0.97		Black / African American	4.17	0.67
	Latino / Hispanic	3.69	0.99		Latino / Hispanic	4.14	0.71
Prospection	Caucasian / White	4.09	0.76	Social Connection	Caucasian / White	3.84	0.80
	Asian	3.90	0.80		Asian	3.88	0.75
	Black / African American	4.46	0.59		Black / African American	3.98	0.86
	Latino / Hispanic	4.20	0.74		Latino / Hispanic	3.63	1.12
Purpose & Meaning	Caucasian / White	3.88	1.02	Stress	Caucasian / White	2.50	0.97
	Asian	3.69	1.04		Asian	2.51	0.94
	Black / African American	4.20	0.94		Black / African American	2.33	0.95
	Latino / Hispanic	4.03	0.84		Latino / Hispanic	2.43	1.21
Resilience	Caucasian / White	3.83	0.87				
	Asian	3.66	0.87				
	Black / African American	4.15	0.84				
	Latino / Hispanic	3.85	0.90				

Note: Caucasian / White (809), Asian (77), Black / African American (57), Latino / Hispanic (56).

© BetterUp Inc. 2019

BetterUp

Appendix B: Scale Definitions

© BetterUp Inc. 2019

Operational Definitions

Sub-dimension	Definitions
Emotional Regulation<br>α = .84	The extent to which we regulate our emotions to remain calm and collected. The more emotionally stable we are, the better equipped we are to remain resilient and excel when challenges arise.
Life Satisfaction<br>α = .84	The extent to which we are fulfilled in our lives.
Prospection<br>α = .87	The extent to which we pragmatically think through ways to achieve our future goals. By envisioning desired future states and figuring out how to turn those desires into reality, we are able to enhance personal meaning and motivation.
Purpose & Meaning<br>α = .92	The extent to which we experience a sense of personal meaning associated with what we do at work.
Resilience<br>α = .88	The extent to which we can recover quickly from stressful experiences.
Self-Awareness<br>α = .70	The extent to which we direct our consciousness to focus on the self. We must be aware of our behaviors in order to set strategic personal goals and work towards achieving them.
Self-Efficacy<br>α = .84	The extent to which we believe that we are capable of producing achieving our goals.
Social Connection<br>α = .80	The extent to which we remain close and engaged with important, supportive people in our lives. By maintaining our close personal relationships, we feel a heightened sense of belonging, and we receive social support that enhances our well-being.
Stress<br>α = .89	The extent to which we experience tension as a result of personal and work circumstances.

© BetterUp Inc. 2019
