# Supplementary material for: Time to Change for Mental Health and Well-being via Virtual Professional Coaching: Longitudinal Observational Study
Source: J Med Internet Res. 2021 Jul 5;23(7):e27774. doi: 10.2196/27774 (PMC8406100; doi:10.2196/27774)
Supplement: Multimedia Appendix 2 [file jmir_v23i7e27774_app2.pdf]

````
---
title: "Multi-level modeling evaluating growth across multiple reflection points"
author: Derek Hutchinson
output:
  html_notebook:
    code_folding: hide
---
````

````
```{r load packages and raw data, message=FALSE, warning=FALSE, include=FALSE}
library(tidyverse)
library(stringr)
library(lme4)
library(sjPlot)
library(knitr)
library(bbplot)
library(lmerTest)
library(emmeans)
library(Hmisc)
library(apaTables)
library(lubridate)
library(effsize)
library(RColorBrewer)
library(ggsci)
library(emmeans)

data_raw <- read_csv("DATA\\onboarding_1month_RP_data_Jun_24_2020.csv", guess = 10000) %>%
  select(-matches(c("onemonth_", "manager_growth_"))) %>%
  select_if(~sum(!is.na(.))>0)
```
````

In order to format the data for these analyses I had to separate Onboarding (OB) from Reflection Point (RP), remove the prefix to each variable name, add a dummy variable of "assessment number" with 0 representing OB, 1 RP1, 2 RP2. I also reduced the number of variables to just the ones we care about... I also reverse-coded stress management into stress! I also had to do some other formatting changes.

````
```{r data format}

data <- data_raw %>%
  filter(RP_number < 3) %>%
  filter(!is.na(RP_submitted_at)) %>%
  select(
    user_id,
    onboarding_submitted_at,
    onboarding_thriving_social_connection:onboarding_job_satisfaction,
````
````

```
    onboarding_life_satisfaction:onboarding_psycap_optimism,
    RP_submitted_at,
    RP_thriving_social_connection:RP_job_satisfaction1,
    RP_life_satisfaction:RP_psycap_optimism,
    RP_number,
    total_sesh
  ) %>%
  rename(RP_job_satisfaction = RP_job_satisfaction1,
         RP_organizational_commitment = RP_organizational_commitment1) %>%
  #Janky code to Filter out RPs less than 3 weeks
  mutate(onboarding_submitted_at = as.Date(onboarding_submitted_at, "%m/%d/
%y", tz ="EST"),
         RP_submitted_at = as.Date(RP_submitted_at, "%m/%d/%y", tz = "EST"),
         rp_days = RP_submitted_at - onboarding_submitted_at) %>%
  filter(rp_days> 21) %>%
  select(-rp_days)

data_ob <- data %>%
  select(-matches("RP_")) %>%
  rename_at(vars(starts_with("onboarding_")), list(~sub("onboarding_", "",
.))) %>%
  mutate(assessment_number = 0) %>%
  distinct(user_id, .keep_all = TRUE) #necessary to deal with extra OBs

data_rp <- data %>%
  select(-matches("onboarding_")) %>%
  rename_at(vars(starts_with("RP_")), list(~sub("RP_", "", .))) %>%
  rename(assessment_number = number)


data_ob_rp <- bind_rows(data_rp, data_ob) %>%
  mutate(stress = abs(stress_management - 6)) %>% #reverse code stress
management back to stress
  group_by(user_id) %>%
  mutate(assessments_total = sum(assessment_number)) %>%
  ungroup() %>%
  filter(assessments_total == 3, # Filter those who had OB, RP1, RP2 only
         !(user_id %in% c(51648, 57048))) # these two members have missing
data.


session_count <- data_ob_rp %>%
  distinct(user_id, .keep_all = TRUE) %>%
  count(total_sesh)

test <- data_ob_rp %>%
  count(assessment_number)

#means at each RP so I can create inital graphics.
```

```
RP_means <- data_ob_rp %>%
  group_by(assessment_number) %>%
  summarise_if(is.numeric, mean, na.rm = TRUE) %>%
  ungroup() %>%
  mutate(
    assessment_number = recode(
      assessment_number,
      "0" = "Onboarding",
      "1" = "RP One",
      "2" = "RP Two"
    ),
    assessment_number = factor(assessment_number, levels = c("Onboarding", "RP
One", "RP Two"))
  )

#group means centering to crate bonus graphics

rp_means_centered <- data_ob_rp %>%
  group_by(user_id) %>%
  mutate(
    stress_centered = stress - mean(stress),
    resilience_centered = resilience - mean(resilience),
    planning_centered = thriving_strategic_planning -
mean(thriving_strategic_planning),
    efficacy_centered = self_efficacy  - mean(self_efficacy),
    purpose_centered = purpose_and_meaning - mean(purpose_and_meaning),
    life_sat_centered = life_satisfaction - mean(life_satisfaction),
    emo_reg_centered = thriving_emotional_regulation -
mean(thriving_emotional_regulation),
    aware_centered = self_awareness - mean(self_awareness),
    social_support_centered = thriving_social_connection -
mean(thriving_social_connection)
  ) %>%
  ungroup()  %>%
  group_by(assessment_number) %>%
  summarise_if(is.numeric, mean, na.rm = TRUE) %>%
  ungroup() %>%
  mutate(
    assessment_number = recode_factor(
      assessment_number,
      "0" = "T1",
      "1" = "T2",
      "2" = "T3",
    .ordered = TRUE)
  )

#Formatting Data for Dimension Analysis
```

```
data_ob_rp_dimension_format <- data_ob_rp %>%
  mutate(stress = abs(stress - 6)) %>% #unreverse code stress to make patterns
the same direction.
  pivot_longer(
    c(-user_id,-submitted_at,-assessment_number, -total_sesh),
    names_to = "WPM",
    values_to = "score"
  ) %>%
  filter(
    WPM %in% c(
      "stress",
      "thriving_strategic_planning",
      "self_awareness",
      "self_efficacy",
      "resilience",
      "purpose_and_meaning",
      "thriving_emotional_regulation",
      "life_satisfaction",
      "thriving_social_connection"
    )
  )
```

Testing if there are Differences across WPM sub-dimensions

The purpose here is to see if the trajectories of growth are dependent upon the dimension. Results here suggest that there the gains that individuals experience at each time point depend on WPM dimension.

```{r ANOVA results FULL, echo=TRUE}
dim_full <- lmer(score ~ assessment_number * WPM + (1|user_id), data =
data_ob_rp_dimension_format)

tab_model(dim_full)

anova(dim_full) %>%
  kable()

#ddf = "Kenward-Roger" add this into the anova for proper paper. Takes 30
minutes to run with KR estimation. Numbers are essentially the same.
```

Testing The predictive strength of total session numbers

We can see here that total session number did not predict scores across all sub-dimensions.

```{r}
dim_sesh <- lmer(score ~ total_sesh + (1|user_id), data =
data_ob_rp_dimension_format)

tab_model(dim_sesh)

anova(dim_sesh) %>%
  kable()
```

Stress Models

Stress: Null Model

```{r Stress Null, message=FALSE, warning=FALSE}

sm <- lmer(stress ~ 1 + (1|user_id), data=data_ob_rp)
tab_model(sm)%>%
    return() %$%
    knitr %>%
    asis_output()
```

Stress: RP Number

```{r Stress RP number, message=FALSE, warning=FALSE}
sm <- lmer(stress ~ assessment_number + (1|user_id), data=data_ob_rp)
tab_model(sm)%>%
    return() %$%
    knitr %>%
    asis_output()

ggplot(rp_means_centered, aes(x = assessment_number, y = stress_centered)) +
  geom_point(size =2) +
  geom_line(aes(group =1), colour = "#1380A1", size = 1) +
      ylim(-.30, .2)+
  bbc_style() +
    labs(title="Group Mean Centered Effects",
        subtitle = "Stress Increase Within-Subjects")
```

Emotional Regulation Models

Emotional Regulation: Null Model

````
```{r emo null, message=FALSE, warning=FALSE}

emom <- lmer(thriving_emotional_regulation ~ 1 + (1|user_id), data=data_ob_rp)
tab_model(emom)%>%
    return() %$%
    knitr %>%
    asis_output()
```

### Emotional Regulation: RP Number

```{r emo RP number, message=FALSE, warning=FALSE}
emom <- lmer(thriving_emotional_regulation ~ assessment_number + (1|user_id),
data=data_ob_rp)
tab_model(emom)%>%
    return() %$%
    knitr %>%
    asis_output()

ggplot(rp_means_centered, aes(x = assessment_number, y = emo_reg_centered)) +
  geom_point(size =2) +
  geom_line(aes(group =1), colour = "#1380A1", size = 1) +
     ylim(-.30, .2)+
  bbc_style() +
    labs(title="Group Mean Centered Effects",
       subtitle = "Emotional Regulation Increase Within-Subjects")
```


# Strategic Planning

### Strategic Planning: Null Model


```{r}
spm <- lmer(thriving_strategic_planning ~ 1 + (1|user_id), data=data_ob_rp)
tab_model(spm)%>%
    return() %$%
    knitr %>%
    asis_output()
```


### Strategic Planning: RP Number

```{r message=FALSE, warning=FALSE}
spm <- lmer(thriving_strategic_planning ~ assessment_number + (1|user_id),
data=data_ob_rp)
tab_model(spm)%>%
````
````

```
    return() %$%
    knitr %>%
    asis_output()

ggplot(rp_means_centered, aes(x = assessment_number, y = planning_centered)) +
  geom_point(size =2) +
  geom_line(aes(group =1), colour = "#1380A1", size = 1) +
     ylim(-.30, .2)+
  bbc_style() +
    labs(title="Group Mean Centered Effects",
       subtitle = "Prospection Increase Within-Subjects")
```

Self-Awareness Models

Self-Awareness: Null Model

```{r}
awarem <- lmer(self_awareness ~ 1 + (1|user_id), data=data_ob_rp)
tab_model(awarem)%>%
    return() %$%
    knitr %>%
    asis_output()
```

Self-Awareness: RP Number

```{r message=FALSE, warning=FALSE}
awarem <- lmer(self_awareness ~ assessment_number + (1|user_id),
data=data_ob_rp)
tab_model(awarem)%>%
    return() %$%
    knitr %>%
    asis_output()

ggplot(rp_means_centered, aes(x = assessment_number, y = aware_centered)) +
  geom_point(size =2) +
  geom_line(aes(group =1), colour = "#1380A1", size = 1) +
     ylim(-.30, .2)+
  bbc_style() +
    labs(title="Group Mean Centered Effects",
       subtitle = "Self-Awareness Increase Within-Subjects")
```

Self-Efficacy

````
### Self-Efficacy: Null Model


```{r}
sem <- lmer(self_efficacy ~ 1 + (1|user_id), data=data_ob_rp)
tab_model(sem)%>%
    return() %$%
    knitr %>%
    asis_output()
```



### Self-Efficacy: RP Number

```{r message=FALSE, warning=FALSE}
sem <- lmer(self_efficacy ~ assessment_number + (1|user_id), data=data_ob_rp)
tab_model(sem)%>%
    return() %$%
    knitr %>%
    asis_output()


ggplot(rp_means_centered, aes(x = assessment_number, y = efficacy_centered)) +
  geom_point(size =2) +
  geom_line(aes(group =1), colour = "#1380A1", size = 1) +
     ylim(-.30, .2)+
  bbc_style() +
    labs(title="Group Mean Centered Effects",
       subtitle = "Self-Efficacy Increase Within-Subjects")
```



# Resilience Models

### Resilience: Null Model

```{r resilience NULL}
rm <- lmer(resilience ~ 1 + (1|user_id), data=data_ob_rp)
tab_model(rm)%>%
    return() %$%
    knitr %>%
    asis_output()
```

### Resilience: RP Number

```{r resilience RP number, message=FALSE, warning=FALSE}
rm <- lmer(resilience ~ assessment_number + (1|user_id), data=data_ob_rp)
tab_model(rm)%>%
````
````

````
    return() %$%
    knitr %>%
    asis_output()

ggplot(rp_means_centered, aes(x = assessment_number, y = resilience_centered))
+
  geom_point(size =2) +
  geom_line(aes(group =1), colour = "#1380A1", size = 1) +
     ylim(-.30, .2)+
  bbc_style() +
    labs(title="Group Mean Centered Effects",
       subtitle = "Resilience Increase Within-Subjects")
```
````

Purpose and Meaning

Purpose and Meaning: Null Model

````
```{r message=FALSE, warning=FALSE}
purm <- lmer(purpose_and_meaning ~ 1 + (1|user_id), data=data_ob_rp)
tab_model(purm)%>%
    return() %$%
    knitr %>%
    asis_output()
```
````

Purpose and Meaning: RP Number

````
```{r message=FALSE, warning=FALSE}
purm <- lmer(purpose_and_meaning ~ assessment_number + (1|user_id),
data=data_ob_rp)
tab_model(purm)%>%
    return() %$%
    knitr %>%
    asis_output()

ggplot(rp_means_centered, aes(x = assessment_number, y = purpose_centered)) +
  geom_point(size =2) +
  geom_line(aes(group =1), colour = "#1380A1", size = 1) +
     ylim(-.30, .2)+
  bbc_style() +
    labs(title="Group Mean Centered Effects",
       subtitle = "Purpose and meaning Increase Within-Subjects")
```
````

Life Satisfaction
````

Life Satisfaction: Null Model

```{r}
satm <- lmer(life_satisfaction ~ 1 + (1|user_id), data=data_ob_rp)
tab_model(satm)%>%
    return() %$%
    knitr %>%
    asis_output()
```

Life Satisfaction: RP Number

```{r message=FALSE, warning=FALSE}
satm <- lmer(life_satisfaction ~ assessment_number + (1|user_id), 
data=data_ob_rp)
tab_model(satm)%>%
    return() %$%
    knitr %>%
    asis_output()

ggplot(rp_means_centered, aes(x = assessment_number, y = life_sat_centered)) +
  geom_point(size =2) +
  geom_line(aes(group =1), colour = "#1380A1", size = 1) +
     ylim(-.30, .2)+
  bbc_style() +
    labs(title="Group Mean Centered Effects",
       subtitle = "Life Satisfaction Increase Within-Subjects")
```

```{r social thriving, message=FALSE, warning=FALSE}
stm <- lmer(thriving_social_connection ~ assessment_number + (1|user_id), 
data=data_ob_rp)

tab_model(stm )%>%
    return() %$%
    knitr %>%
    asis_output()

ggplot(rp_means_centered, aes(x = assessment_number, y = 
social_support_centered)) +
  geom_point(size =2) +
  geom_line(aes(group =1), colour = "#1380A1", size = 1) +
     ylim(-.30, .2)+
  bbc_style() +
    labs(title="Group Mean Centered Effects",
       subtitle = "Social Connectin Increase Within-Subjects")
```

````
```
````

These are graphics using group mean centered variables.

````
```{r group mean centered graphics}

rp_means_centered_facet <- rp_means_centered %>%
  select(assessment_number, matches("centered")) %>%
  select(-compassion_centered, -agility_centered, -empathy_centered, -
growth_centered, -authenticity_centered, -work_life_centered) %>%
  pivot_longer(-assessment_number, names_to = "dimension", values_to =
"score") %>%
  mutate(dimension = recode_factor(dimension,
      "planning_centered" ="Prospection",
      "aware_centered" = "Self-Awareness",
      "efficacy_centered" = "Self-Efficacy",
      "social_support_centered" = "Social Connection",
      "stress_centered" = "Stress",
      "emo_reg_centered" = "Emotion Regulation",
      "life_sat_centered" = "Life Satisfaction",
      "purpose_centered" = "Purpose & Meaning",
      "resilience_centered" = "Resilience",
      .ordered = TRUE)
  )

ggplot(rp_means_centered_facet, aes(x = assessment_number, y = score)) +
  geom_point(size = 1) +
  geom_line(aes(group =1), linetype = "longdash", size = .50, show.legend =
FALSE) +
  facet_wrap(~(dimension)) +
  ylab("Average Deviation from Subject's Mean") +
  theme_bw()+
  theme(
    strip.text.x = element_text(size = 8.5, face = "bold"),
    axis.title.y = element_text(vjust = 3),
    axis.title.x = element_blank(),
    legend.title = element_blank()
  ) +
    ggsave("OUTPUT\\change_facet.png", height = 5, width = 7, units = "in", bg
= "transparent")
```
````

````
```{r correlation matrix}
````

```
matrix_data <- data_ob_rp %>%
  select(user_id, thriving_emotional_regulation, life_satisfaction,
thriving_strategic_planning, purpose_and_meaning,
         resilience, self_awareness, self_efficacy, stress,
thriving_social_connection) %>%
  rename("Stress" = stress,
         "Emotional Regulation" = thriving_emotional_regulation,
         "Prospection"  = thriving_strategic_planning,
         "Self-Awareness" = self_awareness,
         "Self-Efficacy" = self_efficacy,
         "Resilience" = resilience,
         "Purpose and Meaning" = purpose_and_meaning,
         "Life Satisfaction" = life_satisfaction,
         "Social Connection" = thriving_social_connection) %>%
  group_by(user_id) %>%
  summarise_all(mean) %>%
  ungroup() %>%
  select(-user_id)


raw_matrix <-rcorr(as.matrix(matrix_data))


apa.cor.table(matrix_data, filename = "OUTPUT\\matrix_data.doc", table.number
= 1, landscape = TRUE)
```

```{r model summary}

tab_model(emom, satm, spm)%>%
    return() %$%
    knitr %>%
    asis_output()

tab_model(purm, rm, awarem)%>%
    return() %$%
    knitr %>%
    asis_output()

tab_model(sem, stm, sm)%>%
    return() %$%
    knitr %>%
    asis_output()
```


```{r cohens d Alexis Script, eval=FALSE, include=FALSE}

tmp<-data_ob_rp
```

```
tmp$assessment_number<-as.factor(as.numeric(data_ob_rp$assessment_number))


#Emotional Regulation

cohen.d(tmp$thriving_emotional_regulation[which(tmp$assessment_number=="1")],
        tmp$thriving_emotional_regulation[which(tmp$assessment_number=="0")],
paired = TRUE)

cohen.d(tmp$thriving_emotional_regulation[which(tmp$assessment_number=="2")],
        tmp$thriving_emotional_regulation[which(tmp$assessment_number=="1")],
paired = TRUE)

#Life Satisfaction

cohen.d(tmp$life_satisfaction[which(tmp$assessment_number=="1")],
        tmp$life_satisfaction[which(tmp$assessment_number=="0")], paired =
TRUE)

cohen.d(tmp$life_satisfaction[which(tmp$assessment_number=="2")],
        tmp$life_satisfaction[which(tmp$assessment_number=="1")], paired =
TRUE)


#Strategic Planning

cohen.d(tmp$thriving_strategic_planning[which(tmp$assessment_number=="1")],
        tmp$thriving_strategic_planning[which(tmp$assessment_number=="0")],
paired = TRUE)

cohen.d(tmp$thriving_strategic_planning[which(tmp$assessment_number=="2")],
        tmp$thriving_strategic_planning[which(tmp$assessment_number=="1")],
paired = TRUE)


#Purpose and Meaning

cohen.d(tmp$purpose_and_meaning[which(tmp$assessment_number=="1")],
        tmp$purpose_and_meaning[which(tmp$assessment_number=="0")], paired =
TRUE)

cohen.d(tmp$purpose_and_meaning[which(tmp$assessment_number=="2")],
        tmp$purpose_and_meaning[which(tmp$assessment_number=="1")], paired =
TRUE)


#Resilience
```

```
cohen.d(tmp$resilience[which(tmp$assessment_number=="1")],
        tmp$resilience[which(tmp$assessment_number=="0")], paired = TRUE)

cohen.d(tmp$resilience[which(tmp$assessment_number=="2")],
        tmp$resilience[which(tmp$assessment_number=="1")], paired = TRUE)


#Self-Awareness

cohen.d(tmp$self_awareness[which(tmp$assessment_number=="1")],
        tmp$self_awareness[which(tmp$assessment_number=="0")], paired = TRUE)

cohen.d(tmp$self_awareness[which(tmp$assessment_number=="2")],
        tmp$self_awareness[which(tmp$assessment_number=="1")], paired = TRUE)

#Self-Efficacy

cohen.d(tmp$self_efficacy[which(tmp$assessment_number=="1")],
        tmp$self_efficacy[which(tmp$assessment_number=="0")], paired = TRUE)

cohen.d(tmp$self_efficacy[which(tmp$assessment_number=="2")],
        tmp$self_efficacy[which(tmp$assessment_number=="1")], paired = TRUE)

#Stress

cohen.d(tmp$stress[which(tmp$assessment_number=="1")],
        tmp$stress[which(tmp$assessment_number=="0")], paired = TRUE)

cohen.d(tmp$stress[which(tmp$assessment_number=="2")],
        tmp$stress[which(tmp$assessment_number=="1")], paired = TRUE)


#Social Connection
cohen.d(tmp$thriving_social_connection[which(tmp$assessment_number=="1")],
        tmp$thriving_social_connection[which(tmp$assessment_number=="0")],
paired = TRUE)

cohen.d(tmp$thriving_social_connection[which(tmp$assessment_number=="2")],
        tmp$thriving_social_connection[which(tmp$assessment_number=="1")],
paired = TRUE)
```

Post-Hoc Analyses

```{r}
#Emotional Regulation
ereg <- lmer(thriving_emotional_regulation ~ assessment_number + (1|user_id),
data=tmp)
```

````
ereg.emm<-emmeans(ereg, list(pairwise ~ assessment_number), adjust = "tukey")
ereg.emm
```

```{r}
#stress
stress <- lmer(stress ~ assessment_number + (1|user_id), data=tmp)
stress.s<-emmeans(stress, list(pairwise ~ assessment_number), adjust =
"tukey")
stress.s
```

```{r}
#prospection
sp <- lmer(thriving_strategic_planning ~ assessment_number + (1|user_id),
data=tmp)
sp.s<-emmeans(sp, list(pairwise ~ assessment_number), adjust = "tukey")
sp.s
```

```{r}
#self awareness
selfaware <- lmer(self_awareness ~ assessment_number + (1|user_id), data=tmp)
selfaware.s<-emmeans(selfaware, list(pairwise ~ assessment_number), adjust =
"tukey")
selfaware.s
```

```{r}
#resilience
res <- lmer(resilience ~ assessment_number + (1|user_id), data=tmp)
res.s<-emmeans(res, list(pairwise ~ assessment_number), adjust = "tukey")
res.s
```

```{r}
#purpose & meaning
pm <- lmer(purpose_and_meaning ~ assessment_number + (1|user_id), data=tmp)
pm.s<-emmeans(pm, list(pairwise ~ assessment_number), adjust = "tukey")
pm.s
```

```{r}
#life satisfaction
lifesat <- lmer(life_satisfaction ~ assessment_number + (1|user_id), data=tmp)
lifesat.s<-emmeans(lifesat, list(pairwise ~ assessment_number), adjust =
"tukey")
lifesat.s
```
````
````

````
```{r}
#Self-Efficacy
selfeff <- lmer(self_efficacy ~ assessment_number + (1|user_id), data=tmp)
selfeff.s<-emmeans(selfeff, list(pairwise ~ assessment_number), adjust =
"tukey")
selfeff.s
```

```{r}
#social connection
soconnect <- lmer(thriving_social_connection ~ assessment_number + (1|
user_id), data=tmp)
soconnect.s<-emmeans(soconnect, list(pairwise ~ assessment_number), adjust =
"tukey")
soconnect.s
```
````
